# Supplementary material for: Prevalence and correlates of intimate partner sexual violence among pregnant women in Napak district, Northeastern Uganda
Source: PLOS Glob Public Health. 2024 Feb 1;4(2):e0002286. doi: 10.1371/journal.pgph.0002286 (PMC10833580; doi:10.1371/journal.pgph.0002286)
Supplement: S1 Appendix — (DOCX) [file pgph.0002286.s001.docx]

# S1 Appendix: QUESTIONNAIRE

**Section A: Socio demographic characteristics of the pregnant woman**

| **Number** | **Question** | **Choices** |
| --- | --- | --- |
| 1 | Age in full years | ……………………………… |
| 2 | What is your current marital status? | Married ❒ Cohabiting ❒ Other ❒ |
| 3 | For how long have you been in a marital relationship? | Less than five years ❒ More than five years ❒ |
| 4 | To what religious denomination do you belong? | Catholic ❒ Christian (Pentecostal) ❒ Muslim ❒ Other……… |
| 5 | Are you formally educated? | Yes ❒ No ❒ |
| 6 | If yes, to what level? | Primary ❒ Secondary ❒ Post-secondary ❒ |
| 7 | Are you currently employed? | Yes ❒ No ❒ |

**Section B: Intimate partner sexual violence**

|  | **Question** | **Choices** |
| --- | --- | --- |
| **8** | Since conception of the current pregnancy, has you spouse/intimate partner coerced you or made unwanted sexual advances to you? | Yes ❒ No ❒ |
| **9** | Since conception of your current pregnancy, has your husband denied you chance to use measures that can protect you from sexually transmitted infections, during intercourse? | Yes ❒ No ❒ |
| **10** | Since conception of the current pregnancy, has your husband/spouse/intimate partner ever forcefully had sexual intercourse with you? | Yes ❒ No ❒ |
| **11** | If yes above, how many times has that happened during this pregnancy? | Once ❒ Twice ❒ Thrice ❒ More than thrice ❒ |

**Section C: Intrapersonal Characteristics**

|  | **Question** | **Choices** |
| --- | --- | --- |
| **12** | Do you have any form of disabilities? | Yes ❒ No ❒ |
| **13** | If yes, what form of disability do you have? | Physical disability ❒ Sensory disability ❒ |
| **14** | In what trimester are you currently? | Second trimester ❒ Third trimester ❒ |
| **15** | Since conception, have you used any substances of abuse? | Yes ❒ No ❒ |
| **16** | If yes, which ones have you used so far | Illicit substances ❒ Non-illicit ❒ Both ❒ |
| **17** | If illicit, which ones have you used? | Marijuana ❒ Khat ❒ Other…………………. |
|  | If non illicit, which ones have you used? | Alcohol ❒ Tobacco ❒ Other…………… |
| **17** | How many pregnancies have you carried so far? | One ❒ Two ❒ Three ❒ More than three ❒ |
| **18** | Have you ever experienced any intimate partner sexual violence in any of those pregnancies? | Yes ❒ No ❒ |
| **19** | Which form of intimate partner sexual violence was it? | Emotional ❒ Physical ❒ Sexual ❒ |
| **20** | How many living children do you currently have? | One ❒ Two ❒ Three ❒ More than three ❒ |
| **21** | Did you plan to have the current pregnancy? | Yes ❒ No ❒ |
| **22** | Do you usually communication with your spouse? | Yes ❒ No ❒ |
| **23** | If yes, how do you rate your level of communication with your spouse? | High ❒ Moderate ❒ Low ❒ |

**Section D: Spousal Characteristics**

|  | **Question** | **Choices** |
| --- | --- | --- |
| 24 | In what age range is your husband/spouse/intimate partner | Between 18 and 28 years ❒ Between 29 and 39 years ❒  Between 39 and 49 years ❒ More than 49 years ❒ |
| 25 | To what religious denomination does your husband/spouse/intimate partner belong? | Catholic ❒ Christian (Pentecostal) ❒ Muslim ❒  Other………………… |
| 26 | Is your husband/spouse/intimate partner formally educated? | Yes ❒ No ❒ |
| 27 | If yes, to what level? | Primary ❒ Secondary ❒ Post-secondary ❒ |
| 28 | Is your husband/spouse/intimate partner currently employed? | Yes ❒ No ❒ |
| 29 | Does your husband/spouse/intimate partner have a habit of engaging in fights | Yes ❒ No ❒ |
| 30 | Does he have any form of disabilities? | Yes ❒ No ❒ |
| 31 | If yes, what form of disability does he have? | Physical disability ❒ Sensory disability ❒ |
| 32 | Does your husband/spouse/intimate partner use any substances of abuse? | Yes ❒ No ❒ |
| 33 | If yes, which ones does he use | Illicit substances ❒ Non-illicit ❒ Both ❒ |
| 34 | If illicit, which ones does he use? | Marijuana ❒ Khat ❒ Other…………………. |
| 35 | If non illicit, which ones have you used? | Alcohol ❒ Tobacco ❒ Other…………… |
| 36 | Since conception, have you ever stayed with spouse’s parents? | Yes ❒ No ❒ |
| 37 | Has your husband/spouse/intimate partner officially brought dowry your parents? | Yes ❒ No ❒ |
| 38 | Does your husband/spouse/intimate partner participate in gambling like sports betting or playing cards? | Yes ❒ No ❒ |
| 39 | Does your spouse allow you to make your own decisions? | Yes ❒ No ❒ |
| 40 | Does your spouse allow you to go somewhere, at your own accord, without consequences? | Yes ❒ No ❒ |
| 41 | Does your spouse allow you to go to talk with your neighbors or visit friends? | Yes ❒ No ❒ |
| 42 | Has your husband/spouse/intimate partner ever physically abused you? | Yes ❒ No ❒ |
| 43 | Does your husband have a preference for a particular gender of children? | Yes ❒ No ❒ |
| 44 | If yes, which gender does he currently prefer? | Female ❒ Male ❒ |

**Section E: Societal Characteristics**

|  | **Question** | **Choices** |
| --- | --- | --- |
| 45 | In this society, intimate partner sexual violence is considered to be normal | Strongly disagree ❒ Disagree ❒ Undecided ❒  Agree ❒ Strongly Agree ❒ |
| 46 | In our society, a man has a right to have sex with his wife any time they desire, irrespective of hesitance from the wife | Strongly disagree ❒ Disagree ❒ Undecided ❒  Agree ❒ Strongly Agree ❒ |
| 47 | Having sex during pregnancy is highly regarded in this society? | Strongly disagree ❒ Disagree ❒ Undecided ❒  Agree ❒ Strongly Agree ❒ |
| 48 | Marital rape is not considered a grave crime traditionally, even during pregnancy? | Strongly disagree ❒ Disagree ❒ Undecided ❒  Agree ❒ Strongly Agree ❒ |
| 49 | A woman should be ready to have sex at any time, according to culture? | Strongly disagree ❒ Disagree ❒ Undecided ❒  Agree ❒ Strongly Agree ❒ |
| 50 | Does anyone in this society provide you with emotional support? | Strongly disagree ❒ Disagree ❒ Undecided  Agree ❒ Strongly Agree ❒ |
| 51 | A pregnant woman that is sexually violated is not allowed to report to the elders, it is shameful | Strongly disagree ❒ Disagree ❒ Undecided ❒  Agree ❒ Strongly Agree ❒ |
