# Supplementary material for: Prevalence and correlates of intimate partner sexual violence among pregnant women in Napak district, Northeastern Uganda
Source: PLOS Glob Public Health. 2024 Feb 1;4(2):e0002286. doi: 10.1371/journal.pgph.0002286 (PMC10833580; doi:10.1371/journal.pgph.0002286)
Supplement: Attachment — Submitted filename: PGPH-D-23-01429_R1 R2 28Nov2023.pdf [file pgph.0002286.s004.pdf]

PLOS Global Public Health

Prevalence and Correlates of Intimate Partner Sexual Violence among Pregnant Women in Napak District, Northeastern Uganda

--Manuscript Draft--

**Manuscript Number:**	PGPH-D-23-01429R1
**Article Type:**	Research Article
**Full Title:**	Prevalence and Correlates of Intimate Partner Sexual Violence among Pregnant Women in Napak District, Northeastern Uganda
**Short Title:**	Prevalence and Correlates of Intimate Partner Sexual Violence in Uganda
**Corresponding Author:**	Ivan Mugisha Taremwa, MSc<br>Clarke International University<br>Kampala, UGANDA
**Order of Authors:**	Godfrey Patrick Amodoi, MSc
	Ivan Mugisha Taremwa, MSc
	Joan Nakakande, MSc
	Pardon Akugizibwe, MSc
	Samuel Mugambe, MSc
	Miisa Nanyingi, PhD
**Keywords:**	Intimate partner sexual violence , pregnant women, Uganda
**Abstract:**	Intimate partner sexual violence (IPSV) during pregnancy is of key reproductive health concern as it is associated with various risks linked to severe intrapartum complications. This study assessed the prevalence and the correlates of intimate partner sexual violence among pregnant women in Napak district, Northeastern Uganda.<br>This was an analytical cross-sectional study conducted among 284 pregnant women who were obtained by systematic sampling in Napak district in the months of November and December, 2020. A structured questionnaire was used to collect the data, and this was analyzed using STATA version 15. The correlates of IPSV were determined at a multivariable level using a Poisson regression model with robust variance at the individual, relationship, and societal levels of the socio-ecological model.<br>The study enrolled 284 pregnant women of whom, 65.5% were aged between 18 and 28 years and 62.0% were not formally educated. Also, 56% of the pregnant women had experienced IPSV during their current pregnancies, the most prevalent form (35.6%) being unwanted sexual advances. Factors of women being in their third or subsequent pregnancies, primiparity, women who rated their communication with their partners as low, being in a relationship for less than five years, lower age of the spouse, and a lower level of education (primary) among women showed a statistical association with IPSV.<br>This study reports a high prevalence of IPSV during pregnancy, and it was associated with individual, relationship and societal factors. Based on this, concerted efforts through sensitization on the dangers of IPSV are required to avert the practice.
**Additional Information:**	
**Question**	**Response**
**Financial Disclosure**<br><br>Enter a financial disclosure statement that describes the sources of funding for the work included in this submission and the	None to disclose A statement can be given mentioning that no funding for this study has been received.

Powered by Editorial Manager® and ProduXion Manager® from Aries Systems Corporation

role the funder(s) played. This includes grants and any commercial funding of the work or authors.<br><br>This statement will be typeset if the manuscript is accepted for publication.<br><br>*Please review the submission guidelines and the instructions link below for detailed requirements and guidance.*	
**Competing Interests**<br><br>On behalf of all authors, disclose any competing interests that could be perceived to bias this work.<br><br>This statement will be typeset if the manuscript is accepted for publication.<br><br>*Please review the instructions link below and PLOS Global Public Health's competing interests policy to determine what information must be disclosed at submission.*	None to disclose A statement mentioning that there is no conflict of interest among authors, would be better.
**Data Availability**<br><br>Before publication, Authors are required to make fully available and without restriction all data underlying their findings. Please see our PLOS Data Policy page for detailed information on this policy.<br><br>A **Data Availability Statement**, detailing where the data can be accessed, is required at first submission. Insert your Data Availability Statement in the box below.<br><br>Please see the data reporting section of our submission guidelines for instructions on what you need to include in your Data	All relevant data are within the paper and are available without any restrictions.

Availability Statement.<br><br>This statement will be typeset if the manuscript is accepted for publication.<br><br>*PLOS allows rare exemptions to address legal and ethical concerns. If you have legal or ethical restrictions, please use the box below to detail these in full sentences for the Journal team to consider.*	

Powered by Editorial Manager® and ProduXion Manager® from Aries Systems Corporation

Manuscript

Click here to access/download;Manuscript;Intimate Partner Sexual Violence in Uganda.docx

Prevalence and Correlates of Intimate Partner Sexual Violence among Pregnant Women in Napak District, Northeastern Uganda

Author names and Affiliations

Godfrey Patrick Amodoi[1], Ivan Mugisha Taremwa[2], Joan Nakakande[1,3], Pardon Akugizibwe[4,5], Samuel Mugambe[1, 2] Miisa Nanyingi[1]

[1]Faculty of Health Sciences, Uganda Martyrs University, Kampala, Uganda

[2]Institute of Allied Health Sciences, Clarke International University, Kampala, Uganda

[3]Department of Community Health and Research, AMBSO, Kampala, Uganda.

[4]Institute of Public Health and Management, Clarke International University, Kampala, Uganda.

[5]Faculty of Health Sciences, Mountains of the Moon University, Fort Portal, Uganda

Authors' full names, institutional addresses, and email addresses

Godfrey Patrick Amodoi

Uganda Martyrs University, P.O Box 5498, Kampala, Uganda

Email: amodfrey@gmail.com

Ivan Mugisha Taremwa

Clarke International University, P.O Box 7782, Kampala, Uganda

E-mail: imugisha@ymail.com

Joan Nakakande

Uganda Martyrs University, P.O Box 5498, Kampala, Uganda

Email: joannakakande2@gmail.com

Pardon Akugizibwe

Clarke International University, P.O Box 7782, Kampala, Uganda

E-mail: akupardon@gmail.com

Samuel Mugambe

Clarke International University, P.O Box 7782, Kampala, Uganda

E-mail: smugambe92@gmail.com

Miisa Nanyingi
Uganda Martyrs University, P.O Box 5498, Kampala, Uganda
Email: mnanyingi@umu.ac.ug

Abstract

Intimate partner sexual violence (IPSV) during pregnancy is of key reproductive health concern as it is associated with various risks linked to severe intrapartum complications. This study assessed the prevalence and the correlates of intimate partner sexual violence among pregnant women in Napak district, Northeastern Uganda.

This was an analytical cross-sectional study conducted among 284 pregnant women who were obtained by systematic sampling in Napak district in the months of November and December, 2020. A structured questionnaire was used to collect the data, and this was analyzed using STATA version 15. The correlates of IPSV were determined at a multivariable level using a Poisson regression model with robust variance at the individual, relationship, and societal levels of the socio-ecological model.

The study enrolled 284 pregnant women of whom, 65.5% were aged between 18 and 28 years and 62.0% were not formally educated. Also, 56% of the pregnant women had experienced IPSV during their current pregnancies, the most prevalent form (63.3%) being unwanted sexual advances. Factors of women being in their third or subsequent pregnancies, primiparity, women who rated their communication with their partners as low, being in a relationship for less than five years, lower age of the spouse, and a lower level of education (primary) among women showed a statistical association with IPSV.

This study reports a high prevalence of IPSV during pregnancy, and it was associated with individual, relationship and societal factors. Based on this, concerted efforts through sensitization on the dangers of IPSV are required to avert the practice.

Introduction

Female gender?

Better to give details on what the goals are.

Not clear.

The elimination of all forms of violence against female gender forms a critical component of the Sustainable Development Goals (SDG), particularly SDG-5.2 (1, 2). Despite global concerted efforts to end female gender violence, this SDG target has only taken a human rights stance, yet Intimate Partner Sexual Violence (IPSV) is associated with devastating reproductive health ramifications (3, 4). As per the definition of the World Health Organization, IPSV can take any of the five forms, namely; rape, unwanted sexual advances, denial to use contraception, forced abortion, or denial to use protective measures against sexually transmitted infections (STIs) (5). IPSV is life-threatening as it increases the risk of antepartum depression, which is associated with premature rupture of membranes and preterm births (6, 7). Also, denial to use protective measures against STIs has more delipidating health implications, including increased risk of urinary tract infections and STIs (8) and vertical transmission (9). Moreover, rape during pregnancy is associated with vaginal trauma, pelvic floor dysfunction, dyspareunia, vaginismus, bacterial vaginosis, and vaginitis (3, 4, 10, 11). These adverse outcomes are associated with high- risk pregnancies, intrapartum, and postpartum complications (12, 13).

Whereas various efforts (legal and policy perspectives) have shaped the measures to mitigate IPSV (14, 15), IPSV remains unacceptably high (up to 70%) in Africa (16); and 10% to 30% prevalence in East Africa (17). In Uganda, varied reports have indicated IPSV ranging from 27.8% (18) to 70.3% (19). Moreover, sociocultural factors such as forced marriages (19) have been reported in

the northeastern region where Napak district is located. In this region, marriage and childbearing decisions are entrenched in a ritualist attribute. For example, cultural practices such as courtship of wrestling which has been used over time for acceptance and readiness to marry potentiates the risk of forced marriage and IPSV (20). This study determined the prevalence and the correlates of intimate partner sexual violence among pregnant women in Napak district, Northeastern Uganda.

Materials and Methods

Study design, site, and duration

The study used an analytical cross-sectional survey design that was based on a positivist approach, which states that "the truth is out and requires that numerical data be collected and analyzed prior to making deductions that may agree or disagree with the stated truth" (21). The study was conducted in Napak district located in Karamoja region of inter-clan and cross-border violent cattle raids located in Northeastern Uganda. Napak district is bordered by Abim District to the northwest, Kotido District to the north, Moroto District to the northeast and east, Nakapiripirit District to the southeast, Katakwi District to the south, Amuria District to the southwest and Otuke District to the west. Napak district is comprised of six sub-counties, namely; Iriiri, Lokopo, Lopei, Lotome, Matany, and Ngoleriet sub-counties. The district has one Private-Not-For-Profit (PNFP) hospital, five health center IIIs, and five health center IIs. The PNFP hospital and all health center IIIs provide maternal services including antenatal, maternity, and child care delivery services. The study was conducted from 30th November 2020 to 30th December 2020.

Study population and size

The study population comprised pregnant women (16-49 years) who were between 8 to 36 weeks of gestation. The gestation period was guided by the Ministry of Health, Uganda in adherence to the World Health Organization recommendation of the four antenatal care visits (22, 23). Pregnant

women within the first trimester tend to think of being pregnant after noticeable pregnancy-related symptoms and serial missed menstrual periods, and this may coincide with the pregnancy confirmation test performed as part of the first trimester antenatal assessment. On the other hand, week 36 of gestation is the last visit within the third trimester, and at this time, some expecting mothers in the fourth antenatal schedule (≥36weeks) may experience gestational discomforts in preparation for labor. The study presumed that within the studied period, any incidence of IPSV could be satisfactorily remembered.

The sample size was guided by the target population size in Napak district being less than 10,000 as assessed by the district health reports for the financial report 2020/2021. On this premise, a sample size formula derived by Daniel (24) that incorporates a population correction factor was used. Considering a proportion of 1082 pregnant women seeking antenatal care in Napak district, and correcting for non-response; a total of 284 pregnant women were considered for this study.

Sampling procedure

As Matany hospital is attended to the majority of the pregnant women in the district; it was purposively sampled so as not to miss out on the relatively larger pool it serves. Then, the study used a simple random sampling for the health center IIIs. To achieve this, a lottery approach was used in which the names of the five health center IIIs were written on equal-sized pieces of paper, folded, and put in a box. They were ruffled and one picked after each ruffle without replacement. That was done until three of the five papers had been picked, following which they were unfolded to reveal the names of the health centers to be sampled. At the three health centre IIIs, pregnant women were non-randomly sampled until the allocated sample size per facility was attained. This approach provided representation, and minimized bias.

Data collection tool and approach

The study used a structured interviewer administered questionnaire (Supplementary Material). This was developed based on the existing literature (6, 12, 13, 16-20), and was reviewed by an expert of sexual gender based violence. Pilot testing od the questionnaire was done among 20 pregnant women at Moroto Regional Referral Hospital, Moroto district and changes were made accordingly to ensure its suitability. Data collection was conducted by the study team and supported by three trained research assistants proficient in the local language (Ngakarimojong).

Study variables and measurement of variables

The dependent variable of this study was IPSV, which was indicated by incidence of any, or all of the following; unwanted sexual advances, rape and denial to use STI preventive measures. Each of the three screening questions of IPSV was an independent indicator of its incidence, as was affirmed by pregnant women. A woman was said to have been sexually violated by her intimate partner if she reported/having had her spouse make sexual advances to her when she was not interested, forced to have sexual intercourse when she did not want to (rape), or denied the use STI preventive measures in the past 2 or 8 months.

The independent variables were individual, relationship, and community factors as per the suppositions of the socio -ecological model (25). The individual factors assessed having and nature of any disabilities, gestation stage, history and specificity of any substance abuse since conception, history and form of intimate partner violence in any previous pregnancies, and communication with the spouse. The relationship characteristics focused on the age, religion, level of education, spouse employment status, spousal history of violence, socio-cultural norms such as bride-price, substance abuse, decision making in the home, and neighborhood attributes. The community

factors included perceived community practices (including a right to have sex with a wife, irrespective of the circumstance at hand, sex consideration during pregnancy, and reporting of improper conduct related to sexual practices in the community). The responses were scored on a Likert scale of ‘strongly agree, agree, undecided, disagree, and strongly disagree. These responses were dichotomized under ‘yes’ (strongly agree, agree) and ‘no’ (undecided, disagree and strongly disagree).

Data management and analysis

Questionnaires were manually checked for completeness, the data therein was coded and entered into EpiInfo^{TM} version 7.0 (*Centre for Disease Control and Prevention, Antlata, US*) and exported to STATA version 15 (*STATA Corporation, College Station, TX*) for analysis. Exploratory data analysis was done to check missing values and outliers. This was supplemented with the analysis of descriptive frequencies, and looking at the outputs, to find out where the totals and frequencies tally. Then, univariate analysis was conducted to analyse the frequencies and percentages of each variable in the studied population. This was followed by bivariable analysis without adjustment for confounders, using a generalized linear model, and modified Poisson with robust variance given that the magnitude of IPSV obtained was more than 10% (26). Multivariate analysis was then considered using the log-binomial model in which all variables that were found to be statistically significant at bivariate analysis were fitted. Backward stepwise Likelihood Ratio (LR) was used to find the significant predictors of IPSV among the pregnant women. The criteria for entering and removing the independent variables from the backward stepwise model was having a $P < 0.05$ in the final model. Statistical significance at this point was set at 5% ($p<0.05$), and the findings were reported using adjusted prevalence ratios along with their corresponding confidence intervals at 95%.

Ethical considerations

The study obtained ethical approval from the research and ethics committee of The AIDS Support Organization (TASO), Kampala (TASOREC/057/2020/-UG-REC-009). Administrative permission was obtained from Napak district health officer, and from each of the participating health facilities. For the adult participants, the study obtained written informed consent, while pregnant women under 18years, these are considered as mature minors, and therefore they provided assent. By the research regulations in Uganda, such are allowed to offer informed consent solely, without seeking the parent or guardians consent. This is in reference to the Uganda National Council of Science and Technology (UNCST) guidelines (available at: https://www.uncst.go.ug/details.php?option=smenu&id=13&Downloads.html) subsection 5.8, page 19.

Participation was voluntary and the study ensured maximum confidentiality considering the intricacy of the study topic. Also, the team was mindful of the anticipated emotional discomfort from those victimized. To such, psychosocial support in form of confidential counseling and post-trauma support was offered by trained healthcare workers who were members of the study team, and referrals were considered to a local non-governmental based organization.

Results

The study enrolled 284 pregnant women of whom, the majority (n=186, 65.5%) were young women aged between 18 and 28 years and 62.0% (n=176) were not formally educated. Further, 84.5% (n=240) of the respondents were cohabiting, and 54.2% (n=154) had been in a marital relationship for less than five years. The socio demographic characteristics of the respondents are given in table 1.

Table 1 shows socio demographic characteristics of the pregnant women sampled

Variable, n=284	Frequency (Percentage)
**Age (in full years)**	
Less than 18	8(2.8)
18 to 28	186(65.5)
29 to 39	86(30.3)
40 to 49	4(1.4)
**Current marital status**	
Married	44(15.5)
Cohabiting	240(84.5)
**Duration in marital relationship**	
Less than five years	154(54.2)
More than five years	130(45.8)
**Religious denomination**	
Catholic	202(71.1)
Christian (Pentecostal)	77(27.1)
Muslim	5(1.8)
**Formally educated**	
Yes	108(38.0)
No	176(62.0)
**Level of education**	
Primary	64(59.3)
Secondary	38(35.2)
Post-secondary	6(5.6)
**Currently employed**	
Yes	62(21.8)
No	222(78.2)

Intimate partner sexual violence

There were 35.6% (n=101) of the pregnant women had had unwanted sexual advances made by their spouses since conception of their current pregnancies. Moreover, 16.9% (n=48) of the respondents had been denied chance to use STIs protection measures. Also, 22.9% (n=65) of the respondents had had forced sexual intercourse, as shown in table 2.

Table 2 shows intimate partner sexual violence

Variable	Frequency (Percentage)
**Unwanted sexual advances**	
Yes	101(35.6)
No	183(64.4)
**Denied chance to use measures of protection from STI**	
Yes	48(16.9)
No	236(83.1)
**Forced sexual intercourse**	
Yes	65(22.9)
No	219(77.1)
**Frequency of rape pregnancy**	
Once	28(43.1)
Twice	29(44.6)
Thrice	6(9.2)
More than thrice	2(3.1)

Multivariate logistic regression analysis of the correlates of IPSV

Women in their second pregnancy had a 21.5% lower prevalence of IPSV (aPR=0.785, 95%CI: 0.684-0.901, p=0.029) than those in their third or subsequent pregnancy. Similarly, women who had one pregnancy (para-one or primipara) had a 25% lower prevalence of IPSV (APR=0.750, 95% CI: 0.571-0.985, p=0.039) than women who had none (nulliparity). More, women who rated their communication with their partners as high had a 45% lower prevalence of IPSV (aPR=0.555, 95%CI: 0.379-0.810, p=0.002) than those who rated it as low. Women in relationships lasting less than five years, on the other hand, had a 1.53 times higher prevalence of IPSV when compared to those in relationships lasting more than five years. IPSV prevalence decreased with increasing age of the spouse from 18 to 28 years (aPR=9.94, 95%CI: 1.477-66.85, p=0.018); 29 to 39 years (aPR=9.385, 95%CI: 1.398-14.963, p=0.021); and 40 to 49 years (aPR=7.954, 95%CI:1.165-54.286, p=0.034) compared to older spouses over 49 years. Also, spouses with a higher level of education (secondary) had a 30% lower prevalence of IPSV (aPR=0.669, 95%CI: 0.537-0.833,

p=0.001), while women with a lower level of education (primary) had a 2.93 higher prevalence of IPSV (aPR=2.927, 95%CI: 1.211-7.075, p=0.017) than those with a higher level of education (secondary), as shown in table 3.

Table 3 showing the multivariable logistic regression analysis of IPSV among respondents

Variable	Pregnancy IPSV		cPR (CI at 95%)	P value	aPR (CI at 95%)	P value
	Experienced n = 158	Not experienced n = 126				
**Gravidity**						
One	33(62.3%)	20(37.7%)	1.325 (0.986 - 1.780)	0.062	0.813 (0.703 - 0.941)	0.066
Two	47(65.3%)	25(34.7%)	1.389 (1.063 - 1.815)	**0.016**	0.785 (0.684 - 0.901)	**0.029**
Three	31(52.5%)	28(47.5%)	1.118 (0.812 - 1.539)	0.494	0.894 (0.787 - 1.015)	0.498
More than three	47(47.0%)	53(53.0%)	1.000		1.000	
**Parity**						
One	41(51.9%)	38(48.1%)	0.753 (0.572 - 0.989)	**0.042**	0.750 (0.571 -0.985)	**0.039**
Two	34(60.7%)	22(39.3%)	0.880 (0.670 - 1.156)	0.359	0.874 (0.658 - 1.161)	0.353
Three	14(66.7%)	7(33.3%)	0.967 (0.682 - 1.369)	0.849	0.980 (0.689 - 1.393)	0.910
More than three	29(41.4%)	41(58.6%)	0.601 (0.433 - 0.834)	**0.002**	0.599 (0.426 - 0.843)	**0.003**
None	40(69.0%)	18(31.0%)	1.000		1.000	
**Level of communication with spouse**						
High	34(37.8%)	56(62.2%)	0.543 (0.372 - 0.793)	**0.002**	0.555 (0.379 - 0.810)	**0.002**
Moderate	106(62.7%)	63(37.3%)	0.902 (0.672 - 1.210)	0.490	0.926 (0.687 - 1.249)	0.616
Low	16(69.6%)	7(30.4%)	1.000		1.000	
**Duration in marital relationship**						
Less than five years	101(65.6%)	53(34.4%)	1.496 (1.194 - 1.874)	**<0.001**	1.533 (1.210 - 1.941)	**<0.001**
More than five years	57(43.8%)	73(56.2%)	1.000		1.000	
**Age range of spouse**						
Between 18 and 28 years	57(62.0%)	35(38.0%)	9.913 (1.476 - 66.574)	**0.018**	9.944 (1.477 - 66.950)	**0.018**
Between 29 and 39 years	78(59.1%)	54(40.9%)	9.455 (1.410 - 63.404)	**0.021**	9.383 (1.398 - 14.963)	**0.021**
Between 40 and 49 years	22(50.0%)	22(50.0%)	8.000 (1.172 - 54.600)	**0.034**	7.954 (1.165 - 54.286)	**0.034**
More than 49 years	1(6.3%)	15(93.8%)	1.000		1.000	
**Spouse formally educated**						
Yes	58(47.2%)	65(52.8%)	0.759 (0.608 - 0.948)	**0.015**	0.669 (0.537 - 0.833)	**<0.001**
No	100(62.1%)	61(37.9%)	1.000		1.000	
**Level of education of woman**						
Primary	30(62.5%)	18(37.5%)	2.656 (1.097 - 6.433)	**0.030**	2.927 (1.211 - 7.075)	**0.017**

Secondary	24(41.4%)	34(58.6%	1.759 (0.708 - 4.369)	0.224	1.662 (0.668 - 4.134)	0.274
Post-secondary	4(23.5%)	13(76.5%)	1.000		1.000	

Discussion

IPSV arguably poses a subtle challenge of reproductive health as it attributes towards various life-threatening complications, namely; postpartum hemorrhage, sepsis, complications of childbirth, and fetomaternal mortality (2). In this study, the prevalence of IPSV was 56% (n = 158), and this was higher compared to most previous studies, for example 40.8% reported from northern Ethiopia (16), 19.9% in western Ethiopia (27), 44.6% in Nigeria (28), 18.8% in Tanzania (29), 9.2% (30) and 35.3% (31) in Kenya, and 10.6% reported from a previous Ugandan study (18). On the other hand, the obtained prevalence is lower than the 70.3% reported from southwestern Uganda (19). The variations in the reported IPSV attests to its growing and disproportionate burden for varied ethnicities. Contrasting to majority of the studies, this study considered IPSV as defined by the World Health Organization, which includes rape, unwanted sexual advances, denial to use contraception, forced abortion, or denial to use protective measures against sexually transmitted infections (STIs) (5). Also, the studied population is highly patriarchal, and this socio-traditional fabric may as well account for the observed high IPSV prevalence. This suggests an urgent need for concerted ethnical approaches to avert the socio-cultural dynamics that negates reproductive health outcomes.

The study indicated that the prevalent form of IPSV was unwanted sexual advances (N=100, 63.3%), a finding that may suggest no physical, but rather emotional violation. Consistent to previous studies, the associated violence may complicate the gestational process as well as augmenting the parturition (3,4,6,7).

As the prevalence of IPSV was higher in multigravidas, both the physiological and anatomic variances may be considered. It seems multigravidas tend to experience more pregnancy-associated mood changes, and may impair sexual drive. Premised on this, multigravidas tend to be less receptive of their spouse's sexual advances and are thus martially raped. Also, whereas sexual drive reduces in the first and third trimesters among all women; the effect is more exacerbated among multigravida women as they additionally experience other pregnancy complications (32). Relationship for less than five years implied that perhaps the couple had yet cultivated healthy communication between them, which makes dialogue less likely, thus increasing the risk of IPSV. This is consistent with previous reports (33, 34), and emphasizes relationship gaps due to breakdown in communication and negotiations. The age of the spouse was significantly correlated with IPSV, consistent with previous reports (35-37). Young age translated to more IPSV, and this may relate both to hormonal and behavioral attributes. Also, with unemployability and emerging drug as well as substance abuses, it is plausible to find a high IPSV in this age range. From this study, education lessened the risk of IPSV. Consistent with other studies (33, 36), formal education comes with numerous positive attributes including being employed and also possible knowledge about the effects of IPSV. Generally, educated men tend to have relatively less controlling behavior due to the limited or non-embracement of patriarchy and male dominance.

The findings of this study ought to be interpreted in light of some limitations. IPSV was self-reported and this depends on the recall and respondents ability to disclose a rather confidential marital prospect. There are population-specific traditional norms which may affect the study findings. The study did not qualitatively explore on the practice of IPSV, and this definitely leaves a knowledge gap that requires critical attention to eliminate the IPSV practice.

Conclusion

This study reports a high prevalence of IPSV among pregnant women and it was associated with women being in their third or subsequent pregnancies, nulliparity, poor-communication with a partner, being in relationship for less than five years, lower age of the spouse, and a lower level of education. Based on the findings, it is important to sensitize the public on the dangers of IPSV to avert the practice are required. A multipronged approach of health education and male involvement in addressing the IPSV practice are desired.

Acknowledgement

We are grateful to the study participants and research assistants.

References

1. UN Women. Ending violence against women. 2022 https://www.unwomen.org/en/what-we-do/ending-violence-against-women (Accessed June 18, 2023)
2. United Nations. Gender-based violence against women and girls. 2022 Available from https://www.ohchr.org/en/women/gender-based-violence-against-women-and-girls (Accessed August 24, 2023)
3. Clephane K, Wilson MC, Heiman JR, Craig AN, Lorenz T. Sexual violence history predicts changes in vaginal immune parameters during sexual arousal. Brain, behavior, and immunity. 2022 Aug 1;104:171-80.
4. Lohner L, Nigbur L, Klasen C, Witzel I, Garland J, Ondruschka B, Anders S. Vaginal injuries after consensual sexual intercourse—a survey among office-based gynecologists in Hamburg, Germany. Forensic Science, Medicine and Pathology. 2022 Sep;18(3):352-8.
5. WHO. (2021). Violence against women. Available from https://www.who.int/news-room/fact-sheets/detail/violence-against-women
6. Fekadu Dadi A, Miller ER, Mwanri L. Antenatal depression and its association with adverse birth outcomes in low and middle-income countries: a systematic review and meta-analysis. PloS one. 2020 Jan 10;15(1):e0227323.
7. Govender D, Naidoo S, Taylor M. Antenatal and postpartum depression: prevalence and associated risk factors among adolescents' in KwaZulu-Natal, South Africa. Depression research and treatment. 2020 Oct;2020.
8. Hassan MF, Rund N, El-Tohamy O, Moussa M, Ali YZ, Moussa N, Abdelrazik AA, Abdallah EA. Does aerobic vaginitis have adverse pregnancy outcomes? prospective observational study. Infectious diseases in obstetrics and gynecology. 2020 Jan 18;2020.

9. Cerveny L, Murthi P, Staud F. HIV in pregnancy: Mother-to-child transmission, pharmacotherapy, and toxicity. Biochimica et Biophysica Acta (BBA)-Molecular Basis of Disease. 2021 Oct 1;1867(10):166206.
10. Torres-de la Roche LA, Krentel H, Devassy R, de Wilde MS, Leicher L, De Wilde RL. Surgical repair of genital injuries after sexual abuse. GMS Interdisciplinary plastic and reconstructive surgery DGPW. 2019;8.
11. Selai C, Elmalem MS, Chartier-Kastler E, Sassoon N, Hewitt S, Rocha MF, Klitsinari L, Panicker JN. Systematic review exploring the relationship between sexual abuse and lower urinary tract symptoms. International Urogynecology Journal. 2023 Mar;34(3):635-53.
12. Agarwal S, Prasad R, Mantri S, Chandrakar R, Gupta S, Babhulkar V, Srivastav S, Jaiswal A, Wanjari MB. A comprehensive review of intimate partner violence during pregnancy and its adverse effects on maternal and fetal health. Cureus. 2023 May 20;15(5).
13. Kalunga K, Ilunga-Mbaya E, Maroy R, Jonathan YT, Olivier NN. Maternal-Fetal Pregnancy Complications of in Minors Raped with or without Physical Restraint in Eastern Democratic Republic of Congo. Open Journal of Obstetrics and Gynecology. 2023 Jan 16;13(1):1-2.
14. U.S. Department of Health & Human Services. Laws on violence against women. 2018 Accessed on 2/10/2020 from https://www.womenshealth.gov/relationships-and-safety/get-help/laws-violence-against-women
15. World Bank. Ending Violence Against Women And Girls: Global And Regional Trends In Women's Legal Protection Against Domestic Violence And Sexual Harassment. 2018. Accessed from http://pubdocs.worldbank.org/en/140781519943384134/EndingViolenceAgainstWomenandGirlsGBVLawsFeb2018.pdf
16. Berhanie E, Gebregziabher D, Berihu H, Gerezgiher A, Kidane G. Intimate partner violence during pregnancy and adverse birth outcomes: a case-control study. Reproductive health. 2019 Dec;16(1):1-9.
17. Rasch V, Van TN, Nguyen HT, Manongi R, Mushi D, Meyrowitsch DW, Gammeltoft T, Wu CS. Intimate partner violence (IPV): the validity of an IPV screening instrument utilized among pregnant women in Tanzania and Vietnam. PloS one. 2018 Feb 1;13(2):e0190856.
18. Epuitai J, Udho S, Auma AG, Nabirye RC. Intimate partner violence among pregnant women in Uganda. African Journal of Midwifery and Women's Health. 2019 Apr 2;13(2):1-5.
19. Katushabe E, Asiimwe JB, Batwala V. Intimate partner violence disclosure and associated factors among pregnant women attending a city hospital in South-Western Uganda: a cross-sectional study. BMC pregnancy and childbirth. 2022 Jun 13;22(1):484.
20. Achen S, Rwabukwali CB, Atekyereza P. Socio-cultural perceptions of sexuality influencing the sexual and reproductive health of pastoral adolescent girls in Karamoja sub-region in Uganda. Social Sciences & Humanities Open. 2021 Jan 1;4(1):100191.
21. Park YS, Konge L, Artino AR. The positivism paradigm of research. Academic medicine. 2020 May 1;95(5):690-4.
22. Villar J, Bergsjo PW. WHO antenatal care randomized trial: manual for the implementation of the new model. InWHO antenatal care randomized trial: manual for the implementation of the new model 2002 (pp. 37-37).
23. Mugisha C, Odit A, Luzze H. Uganda Clinical Guidelines: National Guidelines on Management of Common Conditions. Uganda: Ministry of Health. 2010:325-78.

24. Daniel W. Biostatistics: A Foundation for analysis in the health sciences, 7th edR Wiley. New York. 1999;141(2).
25. Kafka JM, Moracco KE, Young BR, Taheri C, Graham LM, Macy RJ, Proescholdbell SK. Fatalities related to intimate partner violence: towards a comprehensive perspective. Injury prevention. 2021 Apr 1;27(2):137-44.
26. Martinez BA, Leotti VB, Silva GD, Nunes LN, Machado G, Corbellini LG. Odds ratio or prevalence ratio? An overview of reported statistical methods and appropriateness of interpretations in cross-sectional studies with dichotomous outcomes in veterinary medicine. Frontiers in veterinary science. 2017 Nov 10;4:193.
27. Abebe Abate B, Admassu Wossen B, Tilahun Degfie T. Determinants of intimate partner violence during pregnancy among married women in Abay Chomen district, Western Ethiopia: a community based cross sectional study. BMC women's health. 2016 Dec;16(1):1-8.
28. Orpin J, Papadopoulos C, Puthussery S. The prevalence of domestic violence among pregnant women in Nigeria: a systematic review. Trauma, Violence, & Abuse. 2020 Jan;21(1):3-15.
29. Mahenge B, Stöckl H, Abubakari A, Mbwambo J, Jahn A. Physical, sexual, emotional and economic intimate partner violence and controlling behaviors during pregnancy and postpartum among women in Dar es Salaam, Tanzania. PloS one. 2016 Oct 18;11(10):e0164376.
30. Stiller M, Bärnighausen T, Wilson ML. Intimate partner violence among pregnant women in Kenya: forms, perpetrators and associations. BMC women's health. 2022 Dec;22(1):1-25.
31. Onuonga K, Onguru D, Ogolla S, Mutanyi JA. Intimate partner violence among pregnant women attending prenatal care in Bondo sub- county, Western Kenya. International Journal of Gynecology & Obstetrics. 2023 Jan;160(1):106-12.
32. Mehari MA, Maeruf H, Robles CC, Woldemariam S, Adhena T, Mulugeta M, Haftu A, Hagose H, Kumsa H. Advanced maternal age pregnancy and its adverse obstetrical and perinatal outcomes in Ayder comprehensive specialized hospital, Northern Ethiopia, 2017: a comparative cross-sectional study. BMC pregnancy and childbirth. 2020 Dec;20(1):1-0.
33. Semahegn A, Mengistie B. Domestic violence against women and associated factors in Ethiopia; systematic review. Reproductive health. 2015 Dec;12(1):1-2.
34. Choi EY, Hyun HJ. A predictive model of domestic violence in multicultural families focusing on perpetrator. Asian nursing research. 2016 Sep 1;10(3):213-20.
35. Owaka IO, Nyanchoka MK, Atieli HE. Intimate partner violence in pregnancy among antenatal attendees at health facilities in West Pokot county, Kenya. Pan African Medical Journal. 2017;28(1).
36. Lencha B, Ameya G, Baresa G, Minda Z, Ganfure G. Intimate partner violence and its associated factors among pregnant women in Bale Zone, Southeast Ethiopia: A cross-sectional study. PloS one. 2019 May 1;14(5):e0214962.
37. Barnawi FH. Prevalence and risk factors of domestic violence against women attending a primary care center in Riyadh, Saudi Arabia. Journal of interpersonal violence. 2017 Apr;32(8):1171-86.

366

Manuscript

Click here to access/download;Manuscript;Intimate Partner Sexual Violence in Uganda-R1 (Clean copy).docx

Prevalence and Correlates of Intimate Partner Sexual Violence among Pregnant Women in Napak District, Northeastern Uganda

Author names and Affiliations

Godfrey Patrick Amodoi[1], Ivan Mugisha Taremwa[2], Joan Nakakande[1,3], Pardon Akugizibwe[4,5], Samuel Mugambe[1, 2] Miisa Nanyingi[1]

[1]Faculty of Health Sciences, Uganda Martyrs University, Kampala, Uganda

[2]Institute of Allied Health Sciences, Clarke International University, Kampala, Uganda

[3]Department of Community Health and Research, AMBSO, Kampala, Uganda.

[4]Institute of Public Health and Management, Clarke International University, Kampala, Uganda.

[5]Faculty of Health Sciences, Mountains of the Moon University, Fort Portal, Uganda

Authors' full names, institutional addresses, and email addresses

Godfrey Patrick Amodoi

Uganda Martyrs University, P.O Box 5498, Kampala, Uganda

Email: amodfrey@gmail.com

Ivan Mugisha Taremwa

Clarke International University, P.O Box 7782, Kampala, Uganda

E-mail: imugisha@ymail.com

Joan Nakakande

Uganda Martyrs University, P.O Box 5498, Kampala, Uganda

Email: joannakakande2@gmail.com

Pardon Akugizibwe

Clarke International University, P.O Box 7782, Kampala, Uganda

E-mail: akupardon@gmail.com

Samuel Mugambe

Clarke International University, P.O Box 7782, Kampala, Uganda

E-mail: smugambe92@gmail.com

Miisa Nanyingi
Uganda Martyrs University, P.O Box 5498, Kampala, Uganda
Email: mnanyingi@umu.ac.ug

Abstract

Intimate partner sexual violence (IPSV) during pregnancy is of key reproductive health concern as it is associated with various risks linked to severe intrapartum complications. This study assessed the prevalence and the correlates of intimate partner sexual violence among pregnant women in Napak district, Northeastern Uganda.

This was an analytical cross-sectional study conducted among 284 pregnant women who were obtained by systematic sampling in Napak district in the months of November and December, 2020. A structured questionnaire was used to collect the data, and this was analyzed using STATA version 15. The correlates of IPSV were determined at a multivariable level using a Poisson regression model with robust variance at the individual, relationship, and societal levels of the socio-ecological model.

The study enrolled 284 pregnant women of whom, 65.5% were aged between 18 and 28 years and 62.0% were not formally educated. Also, 56% of the pregnant women had experienced IPSV during their current pregnancies, the most prevalent form (35.6%) being unwanted sexual advances. Factors of women being in their third or subsequent pregnancies, primiparity, women who rated their communication with their partners as low, being in a relationship for less than five years, lower age of the spouse, and a lower level of education (primary) among women showed a statistical association with IPSV.

This study reports a high prevalence of IPSV during pregnancy, and it was associated with individual, relationship and societal factors. Based on this, concerted efforts through sensitization on the dangers of IPSV are required to avert the practice.

Introduction

The elimination of all forms of violence against female gender forms a critical component of the Sustainable Development Goals (SDG), particularly SDG 5 target 2 (1, 2). Despite global concerted efforts to end female gender violence, this SDG target has only taken a human rights stance, yet Intimate Partner Sexual Violence (IPSV) is associated with devastating reproductive health ramifications (3, 4). As per the definition of the World Health Organization, IPSV can take any of the five forms, namely; rape, unwanted sexual advances, denial to use contraception, forced abortion, or denial to use protective measures against sexually transmitted infections (STIs) (5). IPSV is life-threatening as it increases the risk of antepartum depression, which is associated with premature rupture of membranes and preterm births (6, 7). Also, denial to use protective measures against sexually transmitted infections (STIs) has more delipidating health implications, including increased risk of urinary tract infections and STIs (8) and vertical transmission of Human Immunodeficiency Virus (HIV) from the mother to the unborn baby (9). Moreover, rape during pregnancy is associated with vaginal trauma, pelvic floor dysfunction, dyspareunia, vaginismus, bacterial vaginosis, and vaginitis (3, 4, 10, 11). These adverse outcomes are associated with high-risk pregnancies, intrapartum, and postpartum complications (12, 13).

Whereas various efforts (legal and policy perspectives) have shaped the measures to mitigate IPSV (14, 15), IPSV remains unacceptably high (up to 70%) in Africa (16); and 10% to 30% prevalence in East Africa (17). In Uganda, varied reports have indicated IPSV ranging from 27.8% (18) to

70.3% (19). Moreover, sociocultural factors such as forced marriages (19) have been reported in the northeastern region where Napak district is located. In this region, marriage and childbearing decisions are entrenched in a ritualist attribute. For example, cultural practices such as courtship of wrestling which has been used over time for acceptance and readiness to marry potentiates the risk of forced marriage and IPSV (20). This study determined the prevalence and the correlates of intimate partner sexual violence among pregnant women in Napak district, Northeastern Uganda.

Materials and Methods

Study design, site, and duration

The study used an analytical cross-sectional survey design that was based on a positivist approach, which states that "the truth is out and requires that numerical data be collected and analyzed prior to making deductions that may agree or disagree with the stated truth" (21). The study was conducted in Napak district located in Karamoja region of inter-clan and cross-border violent cattle raids located in Northeastern Uganda. Napak district is bordered by Abim District to the northwest, Kotido District to the north, Moroto District to the northeast and east, Nakapiripirit District to the southeast, Katakwi District to the south, Amuria District to the southwest and Otuke District to the west. Napak district is comprised of six sub-counties, namely; Iriiri, Lokopo, Lopei, Lotome, Matany, and Ngoleriet sub-counties. The district has one Private-Not-For-Profit (PNFP) hospital, five health center IIIs, and five health center IIs. The PNFP hospital and all health center IIIs provide maternal services including antenatal, maternity, and child care delivery services. The study was conducted from 30th November 2020 to 30th December 2020.

Study population and size

The study population comprised pregnant women (16-49 years) who were between 8 to 36 weeks of gestation. The ANC visits period was guided by the Ministry of Health, Uganda in adherence

to the World Health Organization recommendation of the eight antenatal care contacts with health system during each pregnancy (22, 23). Pregnant women within the first trimester tend to think of being pregnant after noticeable pregnancy-related symptoms and serial missed menstrual periods, and this may coincide with the pregnancy confirmation test performed as part of the first trimester antenatal assessment. On the other hand, week 36 of gestation is the last visit within the third trimester, and at this time, some expecting mothers in the fourth antenatal schedule (≥36weeks) may experience gestational discomforts in preparation for labor.

The sample size was guided by the target population size in Napak district being less than 10,000 as assessed by the district health reports for the financial report 2020/2021. On this premise, a sample size formula derived by Daniel (24) that incorporates a population correction factor was used. Considering a proportion of 1082 pregnant women seeking antenatal care in Napak district, and correcting for non-response; a total of 284 pregnant women were considered for this study.

Sampling procedure

As Matany hospital is attended by the majority of the pregnant women in the district; it was purposively sampled so as not to miss out on the relatively larger pool it serves. Then, the study used a simple random sampling for the health center IIIs. The sampling frame of pregnant women of 8 to 36 week of gestation. The study presumed that within this period, any incident IPV could be satisfactorily remembered. To achieve this, a lottery approach was used in which the names of the five health center IIIs were written on equal-sized pieces of paper, folded, and put in a box. They were ruffled and one picked after each ruffle without replacement. That was done until three of the five papers had been picked, following which they were unfolded to reveal the names of the health centers to be sampled. At the three health centre IIIs, pregnant women were non-randomly

sampled until the allocated sample size per facility was attained. This approach provided representative sample, and minimized selection bias.

Data collection tool and approach

The study used a structured interviewer administered questionnaire (Supplementary Material). This was developed based on the existing literature (6, 12, 13, 16-20), and was reviewed by an expert of sexual gender based violence. The interviews were conducted on a one-on-one basis, with husbands excluded from participating to ensure candid responses. These interviews took place in a health facility setting during the ANC visits of eligible women with dedicated rooms provided to ensure privacy and confidentiality. A pilot testing the questionnaire was done among 20 pregnant women at Moroto Regional Referral Hospital, Moroto district and changes were made accordingly to ensure its suitability. Data collection was conducted by the study team and supported by three trained research assistants proficient in the local language (Ngakarimojong).

Study variables and measurement of variables

The dependent variable of this study was the experience of IPSV during pregnancy, which was indicated by incidence of any, or all of the following; unwanted sexual advances defined as 'the partner attempted to have sexual contact ignoring the woman's objections, rape was defined as 'the woman was physically forced to have sexual intercourse when she did not want to and denial to use STI preventive measures defines as 'the woman was refused to STI preventive measures such as condom when she needed to use one' (25). Each of the three screening questions of IPSV was an independent indicator of its incidence, as was affirmed by pregnant women. A woman was said to have been sexually violated by her intimate partner if she reported/having had her spouse make sexual advances to her when she indicated that she was not interested, forced to have sexual

intercourse when she did not want to (rape), or denied the use STI preventive measures in the past 2 or 8 months. According to WHO (25), sexual advances made by an intimate partner without the consent or against the will of the other person is a form of intimate partner violence.

The independent variables were individual, relationship, and community factors as per the suppositions of the socio -ecological model (26). The individual factors assessed having and nature of any disabilities, gestation stage, history and specificity of any substance abuse since conception, history and form of intimate partner violence in any previous pregnancies, and communication with the spouse. The relationship characteristics focused on the age, religion, level of education, spouse employment status, spousal history of violence, socio-cultural norms such as bride-price, substance abuse, decision making in the home, and neighborhood attributes. The community factors included perceived community practices (including a right to have sex with a wife, irrespective of the circumstance at hand, sex consideration during pregnancy, and reporting of improper conduct related to sexual practices in the community). The responses were scored on a Likert scale of 'strongly agree, agree, undecided, disagree, and strongly disagree. These responses were dichotomized under 'yes' (strongly agree, agree) and 'no' (undecided, disagree and strongly disagree).

Data management and analysis

Questionnaires were manually checked for completeness, the data therein was coded and entered into EpiInfo^{TM} version 7.0 (*Centre for Disease Control and Prevention*, *Antlata, US*) and exported to STATA version 15 (*STATA Corporation, College Station, TX*) for analysis. Exploratory data analysis was done to check missing values and outliers. This was supplemented with the analysis of descriptive frequencies, and looking at the outputs, to find out where the totals and frequencies tally. Then, univariate analysis was conducted to analyze the frequencies and percentages of each variable in the studied population. This was followed by bivariable analysis without adjustment

for confounders, using a generalized linear model, and modified Poisson with robust variance given that the magnitude of IPSV obtained was more than 10% (27). Bivariate analysis was carried out with independent variables to select the variables for multivariable analysis at a p-value ≤0.20. Multivariate analysis was then considered using the log-binomial model in which all variables that were found to be statistically significant at bivariate analysis were fitted. We identified the correlates of IPSV stopping when all the remaining variables were significant at a 5% (0.05) level of significance. Backward stepwise Likelihood Ratio (LR) was used to find the significant predictors of IPSV among the pregnant women. Statistical significance at this point was set at 5% ($p<0.05$), and the findings were reported using adjusted prevalence ratios along with their corresponding confidence intervals at 95%.

Ethical considerations

The study obtained ethical approval from the research and ethics committee of The AIDS Support Organization (TASO), Kampala (TASOREC/057/2020/-UG-REC-009). Administrative permission was obtained from Napak district health officer, and from each of the participating health facilities. For the adult participants, the study obtained written informed consent, while pregnant women under 18years, these are considered as mature minors, and therefore they provided assent. By the research regulations in Uganda, such are allowed to offer informed consent solely, without seeking the parent or guardians consent. This is in reference to the Uganda National Council of Science and Technology (UNCST) guidelines (available at: https://www.uncst.go.ug/details.php?option=smenu&id=13&Downloads.html) subsection 5.8, page 19.

Participation was voluntary and the study ensured maximum confidentiality considering the intricacy of the study topic. Also, the team was mindful of the anticipated emotional discomfort from those victimized. To such, psychosocial support in form of confidential counseling and post-

trauma support was offered by trained healthcare workers who were members of the study team, and referrals were considered to a local non-governmental based organization.

Results

The study enrolled 284 pregnant women of whom, the majority (n=186, 65.5%) were young women aged between 18 and 28 years and 62.0% (n=176) were not formally educated. Further, 84.5% (n=240) of the respondents were cohabiting, and 54.2% (n=154) had been in a marital relationship for less than five years. The socio demographic characteristics of the respondents are given in table 1.

Table 1 shows socio demographic characteristics of the pregnant women sampled in Napak district, Uganda.

Variable, n=284	Frequency (Percentage)
**Age (in full years)**	
Less than 18	8(2.8)
18 to 28	186(65.5)
29 to 39	86(30.3)
40 to 49	4(1.4)
**Current marital status**	
Married	44(15.5)
Cohabiting	240(84.5)
**Duration in marital relationship**	
Less than five years	154(54.2)
More than five years	130(45.8)
**Religious denomination**	

Catholic	202(71.1)
Christian (Pentecostal)	77(27.1)
Muslim	5(1.8)
**Formally educated**	
Yes	108(38.0)
No	176(62.0)
**Level of education**	
Primary	64(59.3)
Secondary	38(35.2)
Post-secondary	6(5.6)
**Currently employed**	
Yes	62(21.8)
No	222(78.2)

Intimate partner sexual violence

There were 35.6% (n=101) of the pregnant women had had unwanted sexual advances made by their spouses since conception of their current pregnancies. Moreover, 16.9% (n=48) of the respondents had been denied chance to use STIs protection measures. Also, 22.9% (n=65) of the respondents had had forced sexual intercourse, as shown in table 2.

Table 2 shows intimate partner sexual violence among pregnant women in Napak district, Uganda.

Variable	Frequency (Percentage)
**Unwanted sexual advances**	
Yes	101(35.6)

No	183(64.4)
**Denied chance to use measures of protection from STI**	
Yes	48(16.9)
No	236(83.1)
**Forced sexual intercourse**	
Yes	65(22.9)
No	219(77.1)
**Frequency of rape pregnancy**	
Once	28(43.1)
Twice	29(44.6)
Thrice	6(9.2)
More than thrice	2(3.1)

Bivariate logistic regression analysis of the correlates of IPSV among pregnant women in Napak district, Uganda.

The different variables considered showed a varying degree of association as depicted by the bivariate logistic regression analysis (table 3), and variables with a p-value ≤0.2 were then considered for the multivariate logistic regression model.

Table 3 showing the bivariate logistic regression analysis of IPSV among pregnant women in Napak district, Uganda.

Variable (Category)	IPSV		Unadjusted risk ratio (95% CI)	p-values	Adjusted odds ratio (95% CI)	P-values
	No (%)	Yes (%)				

	**n=126**	**n=156**				
**Individual level**						
**Religion**						
Catholic	83 (65.9)	119 (75.3)	1			
Other (Pentecostal/Islam)	43 (34.1)	39 (24.7)	0.63 (0.37-1.05)	**0.082**	0.43(0.22-0.91)	**0.028**
**Level of education**						
No education	72(57.1)	104(65.8)	1			
Primary	29(23.0)	35(22.2)	0.84(0.47 -1.49)	0.541	0.50(0.20-1.26)	0.141
Post-primary	25(19.9)	19(12.0)	0.53(0.27-1.03)	**0.060**	1.31(0.35-4.86)	0.689
**Trimester of pregnancy**						
Second	46(36.5)	70(44.3)	1			
Third	80(63.5)	88(55.7)	0.72(0.45-1.17)	0.185	0.78(0.38-1.66)	0.533
**Pregnancy ever carried**						
one	20(15.9)	33(20.9)	1			
Two	25(19.8)	47(29.8)	1.14(0.54-2.38)	0.720	4.17(0.67-25.75)	0.124
Three	28(22.2)	31(19.5)	0.67(0.32-1.43)	0.300	0.49(0.07-3.53)	0.481
More than three	53(42.1)	47(29.8)	0.54(0.27-1.06)	**0.074**	0.68(0.06-8.18)	0.763
**Number of living children**						
one	38(30.2)	41(26.0)	1			
Two	22(17.5)	34(21.5)	1.43(0.72-2.87)	0.311	6.96(1.52-30.82)	**0.013**
Three	7(5.6)	14(8.9)	1.85(0.68-5.08)	0.231	3.6(0.81-16.38)	0.091
More than three	41(32.5)	29(18.3)	0.66(0.34-1.25)	0.202	5.12(0.55-48.05)	0.153
None	18(14.2)	40(25.3)	2.06(1.01- 4.19)	**0.046**	7.40(1.25-43-67)	**0.027**

**Was pregnancy planned**						
Yes	71(56.4)	77(48.7)	1			
No	55(43.7)	81(51.3)	1.36(0.85-2.17)	**0.200**	0.54(0.23-1.23)	0.147
**Relationship level**						
**Age of partner (years)**						
18-28	35 (27.8)	57(36.1)	1			
29-39	54(42.9)	78(49.4)	0.89(0.51- 1.53)	0.666	1.07(0.42-2.71)	0.880
40-49	22(17.5)	22(13.9)	0.61(0.30-1.27)	0.188	0.22(0.05-0.94)	**0.041**
>49 years	15(11.9)	1(0.6)	0.04(0.01- 0.32)	**0.002**	0.01(0.001-0.112)	**<.001**
**Education level of partner**						
No education	61(48.4)	100(63.3)	1			
Primary level	18(14.3)	30(19.0)	1.02(0.52-1.98)	0.961	0.87(0.34-2.25)	0.781
Post-primary	47(37.3)	28(17.7)	0.36(0.21-0.64)	**<.001**	0.23(0.08-0.64)	**0.005**
**Partner employed**						
Yes	71(56.3)	74(47.4)	1			
No	56(43.7)	47(52.6)	1.43(0.89-2.29)	**0.137**	2.82(1.28-6.17)	**0.010**
**Duration in the relationship**						
< 5 years	53 (42.1)	101 (63.9)	1			
≥ 5years	73 (57.9)	57 (36.1)	0.41 (0.25-0.66)	**<.001**	0.38(0.11-1.33)	0.130
Have you ever experienced IPSV when not pregnant?						
No	115(91.3)	92(58.2)	1			

Yes	11(8.7)	66(41.8)	2.66(3.74-5.02)	**<.001**	11.06(3.77-35.72)	**<.001**
**Level communication with partner**						
High	56(44.4)	34(21.8)				
Moderate	63(50.0)	106(68.0)	2.77(1.63- 4.70)	<.001	2.83(1.32-6.04)	**0.006**
Low	7(5.6)	16(10.2)	3.76(1.41-0.08)	**0.008**	0.38(0.06-2.26)	0.288
**Partner involvement in gambling**						
Yes	15(11.9)	38(24.1)	1			
No	111(88.1)	120(75.9)	0.43(0.22-0.81)	**0.010**	0.44(0.17-1.20)	0.111
**Partner allows visits to friends and neighbors**						
Yes	120(95.3)	144(91.1)	1			
No	6(4.8)	14(8.9)	1.94(o.72-5.21)	**0.186**	0.76(0.13-4.34)	0.761
**Societal level**						
**Partner has gender preferences for children**						
Yes	14(11.1)	29(18.4)	1			
No	112(88.9)	129(81.7)	0.55(0.28-1.10)	**0.094**	omitted	
**Gender preference(n=43)**						
Female	10(71.4)	9(31.0)	1			
Male	4(28.6)	20(69.0)	5.56(1.36-22.56)	**0.016**	5.56(1.36-22.56)	**0.016**
**Marital rape is not considered criminal**						
No	61(48.4)	59(37.3)	1			
Yes	65(51.6)	99(62.7)	1.57(0.98-2.53)	**0.061**	1.38(0.83-2.29)	0.216

**Does anyone provide emotional support?**						
No	30(23.8)	21(13.3)	1			
Yes	96(76.2)	137(86.7)	2.04(1.10-3.77)	0.023	1.80(0.94-3.46)	0.077
A pregnant woman sexually abused is not allowed to report						
No	26(20.6)	18(11.4)				
Yes	100(79.4)	140(88.6)	2.02(1.05-3.89)	**0.035**	1.93(0.95-3.89)	0.065

Multivariate logistic regression analysis of the correlates of IPSV

Women in their second pregnancy had a 21.5% lower prevalence of IPSV (aPR=0.785, 95%CI: 0.684-0.901, p=0.029) than those in their third or subsequent pregnancy. Similarly, women who had one pregnancy (para-one or primipara) had a 25% lower prevalence of IPSV (APR=0.750, 95% CI: 0.571-0.985, p=0.039) than women who had none (nulliparity). More, women who rated their communication with their partners as high had a 45% lower prevalence of IPSV (aPR=0.555, 95%CI: 0.379-0.810, p=0.002) than those who rated it as low. Women in relationships lasting less than five years, on the other hand, had a 1.53 times higher prevalence of IPSV when compared to those in relationships lasting more than five years. IPSV prevalence decreased with increasing age of the spouse from 18 to 28 years (aPR=9.94, 95%CI: 1.477-66.85, p=0.018); 29 to 39 years (aPR=9.385, 95%CI: 1.398-14.963, p=0.021); and 40 to 49 years (aPR=7.954, 95%CI:1.165-54.286, p=0.034) compared to older spouses over 49 years. Also, spouses with a higher level of education (secondary) had a 30% lower prevalence of IPSV (aPR=0.669, 95%CI: 0.537-0.833, p=0.001), while women with a lower level of education (primary) had a 2.93 higher prevalence of

IPSV (aPR=2.927, 95%CI: 1.211-7.075, p=0.017) than those with a higher level of education (secondary), as shown in table 4.

Table 4 showing the multivariable logistic regression analysis of IPSV among pregnant women in Napak district, Uganda.

Variable	Pregnancy IPSV		cPR (CI at 95%)	P value	aPR (CI at 95%)	P value
	Experienced n = 158	Not experienced n = 126				
**Gravidity**						
One	33(62.3%)	20(37.7%)	1.325 (0.986 - 1.780)	0.062	0.813 (0.703 - 0.941)	0.066
Two	47(65.3%)	25(34.7%)	1.389 (1.063 - 1.815)	**0.016**	0.785 (0.684 - 0.901)	**0.029**
Three	31(52.5%)	28(47.5%)	1.118 (0.812 - 1.539)	0.494	0.894 (0.787 - 1.015)	0.498
More than three	47(47.0%)	53(53.0%)	1.000		1.000	
**Parity**						
One	41(51.9%)	38(48.1%)	0.753 (0.572 - 0.989)	**0.042**	0.750 (0.571 -0.985)	**0.039**
Two	34(60.7%)	22(39.3%)	0.880 (0.670 - 1.156)	0.359	0.874 (0.658 - 1.161)	0.353
Three	14(66.7%)	7(33.3%)	0.967 (0.682 - 1.369)	0.849	0.980 (0.689 - 1.393)	0.910
More than three	29(41.4%)	41(58.6%)	0.601 (0.433 - 0.834)	**0.002**	0.599 (0.426 - 0.843)	**0.003**
None	40(69.0%)	18(31.0%)	1.000		1.000	
**Level of communication with spouse**						
High	34(37.8%)	56(62.2%)	0.543 (0.372 - 0.793)	**0.002**	0.555 (0.379 - 0.810)	**0.002**
Moderate	106(62.7%)	63(37.3%)	0.902 (0.672 - 1.210)	0.490	0.926 (0.687 - 1.249)	0.616
Low	16(69.6%)	7(30.4%)	1.000		1.000	
**Duration in marital relationship**						

Less than five years	101(65.6%)	53(34.4%)	1.496 (1.194 - 1.874)	**<0.001**	1.533 (1.210 - 1.941)	**<0.001**
More than five years	57(43.8%)	73(56.2%)	1.000		1.000	
**Age range of spouse**						
Between 18 and 28 years	57(62.0%)	35(38.0%)	9.913 (1.476 - 66.574)	**0.018**	9.944 (1.477 - 66.950)	**0.018**
Between 29 and 39 years	78(59.1%)	54(40.9%)	9.455 (1.410 - 63.404)	**0.021**	9.383 (1.398 - 14.963)	**0.021**
Between 40 and 49 years	22(50.0%)	22(50.0%)	8.000 (1.172 - 54.600)	**0.034**	7.954 (1.165 - 54.286)	**0.034**
More than 49 years	1(6.3%)	15(93.8%)	1.000		1.000	
**Spouse formally educated**						
Yes	58(47.2%)	65(52.8%)	0.759 (0.608 - 0.948)	**0.015**	0.669 (0.537 - 0.833)	**<0.001**
No	100(62.1%)	61(37.9%)	1.000		1.000	
**Level of education of woman**						
Primary	30(62.5%)	18(37.5%)	2.656 (1.097 - 6.433)	**0.030**	2.927 (1.211 - 7.075)	**0.017**
Secondary	24(41.4%)	34(58.6%	1.759 (0.708 - 4.369)	0.224	1.662 (0.668 - 4.134)	0.274
Post-secondary	4(23.5%)	13(76.5%)	1.000		1.000	

Discussion

IPSV arguably poses a subtle challenge of reproductive health as it attributes towards various life-threatening complications, namely; postpartum hemorrhage, sepsis, complications of childbirth, and fetomaternal mortality (2). In this study, the prevalence of IPSV was 56% (n = 158), and this was higher compared to most previous studies, for example 40.8% reported from northern Ethiopia (16), 19.9% in western Ethiopia (28), 44.6% in Nigeria (29), 18.8% in Tanzania (30), 9.2% (31) and 35.3% (32) in Kenya, and 10.6% reported from a previous Ugandan study (18). On the other hand, the obtained prevalence is lower than the 70.3% reported from southwestern Uganda (19). The variations in the reported IPSV attests to its growing and disproportionate burden for varied

ethnicities. Contrasting to majority of the studies, this study considered IPSV as defined by the World Health Organization, which includes rape, unwanted sexual advances, denial to use contraception, forced abortion, or denial to use protective measures against sexually transmitted infections (STIs) (5). Also, the studied population is highly patriarchal, and this socio-traditional fabric may as well account for the observed high IPSV prevalence. This suggests an urgent need for concerted ethnical approaches to avert the socio-cultural dynamics that negates reproductive health outcomes.

The study indicated that the prevalent form of IPSV was unwanted sexual advances (N=101, 35.6%), a finding that may suggest no physical, but rather emotional violation. Consistent to previous studies, the associated violence may complicate the gestational process as well as augmenting the parturition (3,4,6,7).

As the prevalence of IPSV was higher in multigravidas, both the physiological and anatomic variances may be considered. It seems multigravidas tend to experience more pregnancy-associated mood changes, and may impair sexual drive. Premised on this, multigravidas tend to be less receptive of their spouse's sexual advances and are thus martially raped. Also, whereas sexual drive reduces in the first and third trimesters among all women; the effect is more exacerbated among multigravida women as they additionally experience other pregnancy complications (33). Relationship for less than five years implied that perhaps the couple had yet cultivated healthy communication between them, which makes dialogue less likely, thus increasing the risk of IPSV. This is consistent with previous reports (34, 35), and emphasizes relationship gaps due to breakdown in communication and negotiations. The age of the spouse was significantly correlated with IPSV, consistent with previous reports (36-38). Young age translated to more IPSV, and this may relate both to hormonal and behavioral attributes. Also, with unemployability and emerging

drug as well as substance abuses, it is plausible to find a high IPSV in this age range. From this study, education lessened the risk of IPSV. Consistent with other studies (34, 37), formal education comes with numerous positive attributes including being employed and also possible knowledge about the effects of IPSV. Generally, educated men tend to have relatively less controlling behavior due to the limited or non-embracement of patriarchy and male dominance.

The findings of this study ought to be interpreted in light of some limitations. IPSV was self-reported and this depends on the recall and respondents ability to disclose a rather confidential marital prospect. There are population-specific traditional norms which may affect the study findings. The study did not qualitatively explore on the practice of IPSV, and this definitely leaves a knowledge gap that requires critical attention to eliminate the IPSV practice. Also, this study may be limited by the interviewer bias and social desirability responses. More, this study did report on partners'/men's characteristics such as their involvement in risky behavior like alcohol/drug abuse, which would be of great value to understanding the IPSV practice and inform policy.

Conclusion

This study reports a high prevalence of IPSV among pregnant women and it was associated with women being in their third or subsequent pregnancies, nulliparity, poor-communication with a partner, being in relationship for less than five years, lower age of the spouse, and a lower level of education. Based on the findings, it is important to sensitize the public on the dangers of IPSV to avert the practice are required. A multipronged approach of health education and male involvement in addressing the IPSV practice are desired.

Acknowledgement

We are grateful to the study participants and research assistants.

References

1. UN Women. Ending violence against women. 2022 https://www.unwomen.org/en/what-we-do/ending-violence-against-women (Accessed June 18, 2023)
2. United Nations. Gender-based violence against women and girls. 2022 Available from https://www.ohchr.org/en/women/gender-based-violence-against-women-and-girls (Accessed August 24, 2023)
3. Clephane K, Wilson MC, Heiman JR, Craig AN, Lorenz T. Sexual violence history predicts changes in vaginal immune parameters during sexual arousal. Brain, behavior, and immunity. 2022 Aug 1;104:171-80.
4. Lohner L, Nigbur L, Klasen C, Witzel I, Garland J, Ondruschka B, Anders S. Vaginal injuries after consensual sexual intercourse—a survey among office-based gynecologists in Hamburg, Germany. Forensic Science, Medicine and Pathology. 2022 Sep;18(3):352-8.
5. WHO. (2021). Violence against women. Available from https://www.who.int/news-room/fact-sheets/detail/violence-against-women
6. Fekadu Dadi A, Miller ER, Mwanri L. Antenatal depression and its association with adverse birth outcomes in low and middle-income countries: a systematic review and meta-analysis. PloS one. 2020 Jan 10;15(1):e0227323.
7. Govender D, Naidoo S, Taylor M. Antenatal and postpartum depression: prevalence and associated risk factors among adolescents' in KwaZulu-Natal, South Africa. Depression research and treatment. 2020 Oct;2020.
8. Hassan MF, Rund N, El-Tohamy O, Moussa M, Ali YZ, Moussa N, Abdelrazik AA, Abdallah EA. Does aerobic vaginitis have adverse pregnancy outcomes? prospective observational study. Infectious diseases in obstetrics and gynecology. 2020 Jan 18;2020.
9. Cerveny L, Murthi P, Staud F. HIV in pregnancy: Mother-to-child transmission, pharmacotherapy, and toxicity. Biochimica et Biophysica Acta (BBA)-Molecular Basis of Disease. 2021 Oct 1;1867(10):166206.
10. Torres-de la Roche LA, Krentel H, Devassy R, de Wilde MS, Leicher L, De Wilde RL. Surgical repair of genital injuries after sexual abuse. GMS Interdisciplinary plastic and reconstructive surgery DGPW. 2019;8.
11. Selai C, Elmalem MS, Chartier-Kastler E, Sassoon N, Hewitt S, Rocha MF, Klitsinari L, Panicker JN. Systematic review exploring the relationship between sexual abuse and lower urinary tract symptoms. International Urogynecology Journal. 2023 Mar;34(3):635-53.
12. Agarwal S, Prasad R, Mantri S, Chandrakar R, Gupta S, Babhulkar V, Srivastav S, Jaiswal A, Wanjari MB. A comprehensive review of intimate partner violence during pregnancy and its adverse effects on maternal and fetal health. Cureus. 2023 May 20;15(5).
13. Kalunga K, Ilunga-Mbaya E, Maroy R, Jonathan YT, Olivier NN. Maternal-Fetal Pregnancy Complications of in Minors Raped with or without Physical Restraint in Eastern Democratic Republic of Congo. Open Journal of Obstetrics and Gynecology. 2023 Jan 16;13(1):1-2.
14. U.S. Department of Health & Human Services. Laws on violence against women. 2018 Accessed on 2/10/2020 from https://www.womenshealth.gov/relationships-and-safety/get-help/laws-violence-against-women
15. World Bank. Ending Violence Against Women And Girls: Global And Regional Trends In Women's Legal Protection Against Domestic Violence And Sexual Harassment. 2018. Accessed

from http://pubdocs.worldbank.org/en/140781519943384134/EndingViolenceAgainstWomenandGirlsGBVLawsFeb2018.pdf

16. Berhanie E, Gebregziabher D, Berihu H, Gerezgiher A, Kidane G. Intimate partner violence during pregnancy and adverse birth outcomes: a case-control study. Reproductive health. 2019 Dec;16(1):1-9.
17. Rasch V, Van TN, Nguyen HT, Manongi R, Mushi D, Meyrowitsch DW, Gammeltoft T, Wu CS. Intimate partner violence (IPV): the validity of an IPV screening instrument utilized among pregnant women in Tanzania and Vietnam. PloS one. 2018 Feb 1;13(2):e0190856.
18. Epuitai J, Udho S, Auma AG, Nabirye RC. Intimate partner violence among pregnant women in Uganda. African Journal of Midwifery and Women's Health. 2019 Apr 2;13(2):1-5.
19. Katushabe E, Asiimwe JB, Batwala V. Intimate partner violence disclosure and associated factors among pregnant women attending a city hospital in South-Western Uganda: a cross-sectional study. BMC pregnancy and childbirth. 2022 Jun 13;22(1):484.
20. Achen S, Rwabukwali CB, Atekyereza P. Socio-cultural perceptions of sexuality influencing the sexual and reproductive health of pastoral adolescent girls in Karamoja sub-region in Uganda. Social Sciences & Humanities Open. 2021 Jan 1;4(1):100191.
21. Park YS, Konge L, Artino AR. The positivism paradigm of research. Academic medicine. 2020 May 1;95(5):690-4.
22. Tunçalp Ö, Pena-Rosas JP, Lawrie T, Bucagu M, Oladapo OT, Portela A, Gülmezoglu AM. WHO recommendations on antenatal care for a positive pregnancy experience-going beyond survival. Bjog. 2017 May 1;124(6):860-2.
23. Mugisha C, Odit A, Luzze H. Uganda Clinical Guidelines: National Guidelines on Management of Common Conditions. Uganda: Ministry of Health. 2010:325-78.
24. Daniel W. Biostatistics: A Foundation for analysis in the health sciences, 7th edR Wiley. New York. 1999;141(2).
25. World Health Organization. Understanding and addressing violence against women: Intimate partner violence. World Health Organization; 2012.
26. Kafka JM, Moracco KE, Young BR, Taheri C, Graham LM, Macy RJ, Proescholdbell SK. Fatalities related to intimate partner violence: towards a comprehensive perspective. Injury prevention. 2021 Apr 1;27(2):137-44.
27. Martinez BA, Leotti VB, Silva GD, Nunes LN, Machado G, Corbellini LG. Odds ratio or prevalence ratio? An overview of reported statistical methods and appropriateness of interpretations in cross-sectional studies with dichotomous outcomes in veterinary medicine. Frontiers in veterinary science. 2017 Nov 10;4:193.
28. Abebe Abate B, Admassu Wossen B, Tilahun Degfie T. Determinants of intimate partner violence during pregnancy among married women in Abay Chomen district, Western Ethiopia: a community based cross sectional study. BMC women's health. 2016 Dec;16(1):1-8.
29. Orpin J, Papadopoulos C, Puthussery S. The prevalence of domestic violence among pregnant women in Nigeria: a systematic review. Trauma, Violence, & Abuse. 2020 Jan;21(1):3-15.
30. Mahenge B, Stöckl H, Abubakari A, Mbwambo J, Jahn A. Physical, sexual, emotional and economic intimate partner violence and controlling behaviors during pregnancy and postpartum among women in Dar es Salaam, Tanzania. PloS one. 2016 Oct 18;11(10):e0164376.

31. Stiller M, Bärnighausen T, Wilson ML. Intimate partner violence among pregnant women in Kenya: forms, perpetrators and associations. BMC women's health. 2022 Dec;22(1):1-25.
32. Onuonga K, Onguru D, Ogolla S, Mutanyi JA. Intimate partner violence among pregnant women attending prenatal care in Bondo sub- county, Western Kenya. International Journal of Gynecology & Obstetrics. 2023 Jan;160(1):106-12.
33. Mehari MA, Maeruf H, Robles CC, Woldemariam S, Adhena T, Mulugeta M, Haftu A, Hagose H, Kumsa H. Advanced maternal age pregnancy and its adverse obstetrical and perinatal outcomes in Ayder comprehensive specialized hospital, Northern Ethiopia, 2017: a comparative cross-sectional study. BMC pregnancy and childbirth. 2020 Dec;20(1):1-0.
34. Semahegn A, Mengistie B. Domestic violence against women and associated factors in Ethiopia; systematic review. Reproductive health. 2015 Dec;12(1):1-2.
35. Choi EY, Hyun HJ. A predictive model of domestic violence in multicultural families focusing on perpetrator. Asian nursing research. 2016 Sep 1;10(3):213-20.
36. Owaka IO, Nyanchoka MK, Atieli HE. Intimate partner violence in pregnancy among antenatal attendees at health facilities in West Pokot county, Kenya. Pan African Medical Journal. 2017;28(1).
37. Lencha B, Ameya G, Baresa G, Minda Z, Ganfure G. Intimate partner violence and its associated factors among pregnant women in Bale Zone, Southeast Ethiopia: A cross-sectional study. PloS one. 2019 May 1;14(5):e0214962.
38. Barnawi FH. Prevalence and risk factors of domestic violence against women attending a primary care center in Riyadh, Saudi Arabia. Journal of interpersonal violence. 2017 Apr;32(8):1171-86.

List of legends

Supplementary Material_Data Collection Tool

Supporting Information_Research and Ethics Committee Approval

Click here to access/download
Supporting Information
Supplementary Material_Data Collection Tool.docx

Click here to access/download
Supporting Information
REC_Approval_Intimate Partner Sexual Violence.pdf

Prevalence and Correlates of Intimate Partner Sexual Violence among Pregnant Women in Napak District, Northeastern Uganda

Author names and Affiliations

Godfrey Patrick Amodoi[1], Ivan Mugisha Taremwa[2], Joan Nakakande[1,3], Pardon Akugizibwe[4,5], Samuel Mugambe[1, 2] Miisa Nanyingi[1]

[1]Faculty of Health Sciences, Uganda Martyrs University, Kampala, Uganda
[2]Institute of Allied Health Sciences, Clarke International University, Kampala, Uganda
[3]Department of Community Health and Research, AMBSO, Kampala, Uganda.
[4]Institute of Public Health and Management, Clarke International University, Kampala, Uganda.
[5]Faculty of Health Sciences, Mountains of the Moon University, Fort Portal, Uganda

Authors' full names, institutional addresses, and email addresses

Godfrey Patrick Amodoi
Uganda Martyrs University, P.O Box 5498, Kampala, Uganda
Email: amodfrey@gmail.com

Ivan Mugisha Taremwa
Clarke International University, P.O Box 7782, Kampala, Uganda
E-mail: imugisha@ymail.com

Joan Nakakande
Uganda Martyrs University, P.O Box 5498, Kampala, Uganda
Email: joannakakande2@gmail.com

Pardon Akugizibwe
Clarke International University, P.O Box 7782, Kampala, Uganda
E-mail: akupardon@gmail.com

Samuel Mugambe
Clarke International University, P.O Box 7782, Kampala, Uganda
E-mail: smugambe92@gmail.com

Miisa Nanyingi
Uganda Martyrs University, P.O Box 5498, Kampala, Uganda
Email: mnanyingi@umu.ac.ug

Abstract

Intimate partner sexual violence (IPSV) during pregnancy is of key reproductive health concern as it is associated with various risks linked to severe intrapartum complications. This study assessed the prevalence and the correlates of intimate partner sexual violence among pregnant women in Napak district, Northeastern Uganda.

This was an analytical cross-sectional study conducted among 284 pregnant women who were obtained by systematic sampling in Napak district in the months of November and December, 2020. A structured questionnaire was used to collect the data, and this was analyzed using STATA version 15. The correlates of IPSV were determined at a multivariable level using a Poisson regression model with robust variance at the individual, relationship, and societal levels of the socio-ecological model.

The study enrolled 284 pregnant women of whom, 65.5% were aged between 18 and 28 years and 62.0% were not formally educated. Also, 56% of the pregnant women had experienced IPSV during their current pregnancies, the most prevalent form (35.63.3%) being unwanted sexual advances. Factors of women being in their third or subsequent pregnancies, primiparity, women who rated their communication with their partners as low, being in a relationship for less than five years, lower age of the spouse, and a lower level of education (primary) among women showed a statistical association with IPSV.

This study reports a high prevalence of IPSV during pregnancy, and it was associated with individual, relationship and societal factors. Based on this, concerted efforts through sensitization on the dangers of IPSV are required to avert the practice.

Introduction

The elimination of all forms of violence against female gender forms a critical component of the Sustainable Development Goals (SDG), particularly SDG 5 target 2 ~~SDG 5.2~~ (1, 2). Despite global concerted efforts to end female gender violence, this SDG target has only taken a human rights stance, yet Intimate Partner Sexual Violence (IPSV) is associated with devastating reproductive health ramifications (3, 4). As per the definition of the World Health Organization, IPSV can take any of the five forms, namely; rape, unwanted sexual advances, denial to use contraception, forced abortion, or denial to use protective measures against sexually transmitted infections (STIs) (5). IPSV is life-threatening as it increases the risk of antepartum depression, which is associated with premature rupture of membranes and preterm births (6, 7). Also, denial to use protective measures against sexually transmitted infections (STIs) has more delipidating health implications, including increased risk of urinary tract infections and STIs (8) and vertical transmission of Human Immunodeficiency Virus (HIV) from the mother to the unborn baby (9). Moreover, rape during pregnancy is associated with vaginal trauma, pelvic floor dysfunction, dyspareunia, vaginismus, bacterial vaginosis, and vaginitis (3, 4, 10, 11). These adverse outcomes are associated with high-risk pregnancies, intrapartum, and postpartum complications (12, 13).

Whereas various efforts (legal and policy perspectives) have shaped the measures to mitigate IPSV (14, 15), IPSV remains unacceptably high (up to 70%) in Africa (16); and 10% to 30% prevalence in East Africa (17). In Uganda, varied reports have indicated IPSV ranging from 27.8% (18) to

70.3% (19). Moreover, sociocultural factors such as forced marriages (19) have been reported in the northeastern region where Napak district is located. In this region, marriage and childbearing decisions are entrenched in a ritualist attribute. For example, cultural practices such as courtship of wrestling which has been used over time for acceptance and readiness to marry potentiates the risk of forced marriage and IPSV (20). This study determined the prevalence and the correlates of intimate partner sexual violence among pregnant women in Napak district, Northeastern Uganda.

Materials and Methods

Study design, site, and duration

The study used an analytical cross-sectional survey design that was based on a positivist approach, which states that "the truth is out and requires that numerical data be collected and analyzed prior to making deductions that may agree or disagree with the stated truth" (21). The study was conducted in Napak district located in Karamoja region of inter-clan and cross-border violent cattle raids located in Northeastern Uganda. Napak district is bordered by Abim District to the northwest, Kotido District to the north, Moroto District to the northeast and east, Nakapiripirit District to the southeast, Katakwi District to the south, Amuria District to the southwest and Otuke District to the west. Napak district is comprised of six sub-counties, namely; Iriiri, Lokopo, Lopei, Lotome, Matany, and Ngoleriet sub-counties. The district has one Private-Not-For-Profit (PNFP) hospital, five health center IIIs, and five health center IIs. The PNFP hospital and all health center IIIs provide maternal services including antenatal, maternity, and child care delivery services. The study was conducted from 30th November 2020 to 30th December 2020.

Study population and size

The study population comprised pregnant women (16-49 years) who were between 8 to 36 weeks of gestation. ~~The gestation period~~ The ANC visits period was guided by the Ministry of Health,

Uganda in adherence to the World Health Organization recommendation of the eight ~~four~~ antenatal care ~~visits~~ contacts with health system during each pregnancy (22, 23). Pregnant women within the first trimester tend to think of being pregnant after noticeable pregnancy-related symptoms and serial missed menstrual periods, and this may coincide with the pregnancy confirmation test performed as part of the first trimester antenatal assessment. On the other hand, week 36 of gestation is the last visit within the third trimester, and at this time, some expecting mothers in the fourth antenatal schedule (≥36weeks) may experience gestational discomforts in preparation for labor. ~~The study presumed that within the studied period, any incidence of IPSV could be satisfactorily remembered.~~

The sample size was guided by the target population size in Napak district being less than 10,000 as assessed by the district health reports for the financial report 2020/2021. On this premise, a sample size formula derived by Daniel (24) that incorporates a population correction factor was used. Considering a proportion of 1082 pregnant women seeking antenatal care in Napak district, and correcting for non-response; a total of 284 pregnant women were considered for this study.

Sampling procedure

As Matany hospital is attended ~~to~~ by the majority of the ~~by~~ pregnant women in the district; it was purposively sampled so as not to miss out on the relatively larger pool it serves. Then, the study used a simple random sampling for the health center IIIs. The sampling frame of pregnant women of 8 to 36 week of gestation. The study presumed that within this period, any incident IPV could be satisfactorily remembered. To achieve this, a lottery approach was used in which the names of the five health center IIIs were written on equal-sized pieces of paper, folded, and put in a box. They were ruffled and one picked after each ruffle without replacement. That was done until three of the five papers had been picked, following which they were unfolded to reveal the names of the

health centers to be sampled. At the three health centre IIIs, pregnant women were non-randomly sampled until the allocated sample size per facility was attained. This approach provided representative sample, and minimized selection bias.

Data collection tool and approach

The study used a structured interviewer administered questionnaire (Supplementary Material). This was developed based on the existing literature (6, 12, 13, 16-20), and was reviewed by an expert of sexual gender based violence. The interviews were conducted on a one-on-one basis, with husbands excluded from participating to ensure candid responses. These interviews took place in a health facility setting during the ANC visits of eligible women with dedicated rooms provided to ensure privacy and confidentiality. A pilot testing od the questionnaire was done among 20 pregnant women at Moroto Regional Referral Hospital, Moroto district and changes were made accordingly to ensure its suitability. Data collection was conducted by the study team and supported by three trained research assistants proficient in the local language (Ngakarimojong).

Study variables and measurement of variables

The dependent variable of this study was the experience of IPSV during pregnancy, which was indicated by incidence of any, or all of the following; unwanted sexual advances defined as 'the partner attempted to have sexual contact ignoring the woman's objections, rape was defined -as 'the woman was physically forced to have sexual intercourse when she did not want to and denial to use STI preventive measures defines as 'the woman was refused to STI preventive measures such as condom when she needed to use one' (25). Each of the three screening questions of IPSV was an independent indicator of its incidence, as was affirmed by pregnant women. A woman was said to have been sexually violated by her intimate partner if she reported/having had her spouse

make sexual advances to her when she indicated that she was not ~~was not~~ interested, forced to have sexual intercourse when she did not want to (rape), or denied the use STI preventive measures in the past 2 or 8 months. According to WHO (25), sexual advances made by an intimate partner without the consent or against the will of the other person is a form of intimate partner violence.

The independent variables were individual, relationship, and community factors as per the suppositions of the socio -ecological model (26~~5~~). The individual factors assessed having and nature of any disabilities, gestation stage, history and specificity of any substance abuse since conception, history and form of intimate partner violence in any previous pregnancies, and communication with the spouse. The relationship characteristics focused on the age, religion, level of education, spouse employment status, spousal history of violence, socio-cultural norms such as bride-price, substance abuse, decision making in the home, and neighborhood attributes. The community factors included perceived community practices (including a right to have sex with a wife, irrespective of the circumstance at hand, sex consideration during pregnancy, and reporting of improper conduct related to sexual practices in the community). The responses were scored on a Likert scale of ‘strongly agree, agree, undecided, disagree, and strongly disagree. These responses were dichotomized under ‘yes’ (strongly agree, agree) and ‘no’ (undecided, disagree and strongly disagree).

Data management and analysis

Questionnaires were manually checked for completeness, the data therein was coded and entered into EpiInfo^{TM} version 7.0 (*Centre for Disease Control and Prevention, Antlata, US*) and exported to STATA version 15 (*STATA Corporation, College Station, TX*) for analysis. Exploratory data analysis was done to check missing values and outliers. This was supplemented with the analysis of descriptive frequencies, and looking at the outputs, to find out where the totals and frequencies tally. Then, univariate analysis was conducted to analyze the frequencies and percentages of each

variable in the studied population. This was followed by bivariable analysis without adjustment for confounders, using a generalized linear model, and modified Poisson with robust variance given that the magnitude of IPSV obtained was more than 10% (27~~6~~). Bivariate analysis was carried out with independent variables to select the variables for multivariable analysis at a p-value ≤0.20. Multivariate analysis was then considered using the log-binomial model in which all variables that were found to be statistically significant at bivariate analysis were fitted. We identified the correlates of IPSV stopping when all the remaining variables were significant at a 5% (0.05) level of significance. Backward stepwise Likelihood Ratio (LR) was used to find the significant predictors of IPSV among the pregnant women. ~~The criteria for entering and removing the independent variables from the backward stepwise model was having a P < 0.05 in the final model.~~ Statistical significance at this point was set at 5% (p<0.05), and the findings were reported using adjusted prevalence ratios along with their corresponding confidence intervals at 95%.

Ethical considerations

The study obtained ethical approval from the research and ethics committee of The AIDS Support Organization (TASO), Kampala (TASOREC/057/2020/-UG-REC-009). Administrative permission was obtained from Napak district health officer, and from each of the participating health facilities. For the adult participants, the study obtained written informed consent, while pregnant women under 18years, these are considered as mature minors, and therefore they provided assent. By the research regulations in Uganda, such are allowed to offer informed consent solely, without seeking the parent or guardians consent. This is in reference to the Uganda National Council of Science and Technology (UNCST) guidelines (available at: https://www.uncst.go.ug/details.php?option=smenu&id=13&Downloads.html) subsection 5.8, page 19.

Participation was voluntary and the study ensured maximum confidentiality considering the intricacy of the study topic. Also, the team was mindful of the anticipated emotional discomfort

from those victimized. To such, psychosocial support in form of confidential counseling and post-trauma support was offered by trained healthcare workers who were members of the study team, and referrals were considered to a local non-governmental based organization.

Results

The study enrolled 284 pregnant women of whom, the majority (n=186, 65.5%) were young women aged between 18 and 28 years and 62.0% (n=176) were not formally educated. Further, 84.5% (n=240) of the respondents were cohabiting, and 54.2% (n=154) had been in a marital relationship for less than five years. The socio demographic characteristics of the respondents are given in table 1.

Table 1 shows socio demographic characteristics of the pregnant women sampled in Napak district, Uganda.

Variable, n=284	Frequency (Percentage)
**Age (in full years)**	
Less than 18	8(2.8)
18 to 28	186(65.5)
29 to 39	86(30.3)
40 to 49	4(1.4)
**Current marital status**	
Married	44(15.5)
Cohabiting	240(84.5)
**Duration in marital relationship**	
Less than five years	154(54.2)
More than five years	130(45.8)
**Religious denomination**	
Catholic	202(71.1)
Christian (Pentecostal)	77(27.1)
Muslim	5(1.8)
**Formally educated**	
Yes	108(38.0)
No	176(62.0)
**Level of education**	

Primary	64(59.3)
Secondary	38(35.2)
Post-secondary	6(5.6)
**Currently employed**	
Yes	62(21.8)
No	222(78.2)

Intimate partner sexual violence

There were 35.6% (n=101) of the pregnant women had had unwanted sexual advances made by their spouses since conception of their current pregnancies. Moreover, 16.9% (n=48) of the respondents had been denied chance to use STIs protection measures. Also, 22.9% (n=65) of the respondents had had forced sexual intercourse, as shown in table 2.

Table 2 shows intimate partner sexual violence among pregnant women in Napak district, Uganda.

Variable	Frequency (Percentage)
**Unwanted sexual advances**	
Yes	101(35.6)
No	183(64.4)
**Denied chance to use measures of protection from STI**	
Yes	48(16.9)
No	236(83.1)
**Forced sexual intercourse**	
Yes	65(22.9)
No	219(77.1)
**Frequency of rape pregnancy**	
Once	28(43.1)
Twice	29(44.6)
Thrice	6(9.2)
More than thrice	2(3.1)

Bivariate logistic regression analysis of the correlates of IPSV among pregnant women in Napak district, Uganda.

The different variables considered showed a varying degree of association as depicted by the bivariate logistic regression analysis (table 3), and variables with a p-value ≤0.2 were then considered for the multivariate logistic regression model.

Table 3 showing the bivariate logistic regression analysis of IPSV among pregnant women in Napak district, Uganda.

**Variable (Category)**	**IPSV**		**Unadjusted risk ratio (95% CI)**	**p-values**	**Adjusted odds ratio (95% CI)**	**P-values**
	**No (%) n=126**	**Yes (%) n=156**				
**Individual level**						
**Religion**						
Catholic	83 (65.9)	119 (75.3)	1			
Other (Pentecostal/Islam)	43 (34.1)	39 (24.7)	0.63 (0.37-1.05)	**0.082**	0.43(0.22-0.91)	**0.028**
**Level of education**						
No education	72(57.1)	104(65.8)	1			
Primary	29(23.0)	35(22.2)	0.84(0.47 -1.49)	0.541	0.50(0.20-1.26)	0.141
Post-primary	25(19.9)	19(12.0)	0.53(0.27-1.03)	**0.060**	1.31(0.35-4.86)	0.689
**Trimester of pregnancy**						
Second	46(36.5)	70(44.3)	1			
Third	80(63.5)	88(55.7)	0.72(0.45-1.17)	0.185	0.78(0.38-1.66)	0.533
**Pregnancy ever carried**						
one	20(15.9)	33(20.9)	1			
Two	25(19.8)	47(29.8)	1.14(0.54-2.38)	0.720	4.17(0.67-25.75)	0.124
Three	28(22.2)	31(19.5)	0.67(0.32-1.43)	0.300	0.49(0.07-3.53)	0.481
More than three	53(42.1)	47(29.8)	0.54(0.27-1.06)	**0.074**	0.68(0.06-8.18)	0.763
**Number of living children**						
one	38(30.2)	41(26.0)	1			
Two	22(17.5)	34(21.5)	1.43(0.72-2.87)	0.311	6.96(1.52-30.82)	**0.013**
Three	7(5.6)	14(8.9)	1.85(0.68-5.08)	0.231	3.6(0.81-16.38)	0.091
More than three	41(32.5)	29(18.3)	0.66(0.34-1.25)	0.202	5.12(0.55-48.05)	0.153
None	18(14.2)	40(25.3)	2.06(1.01- 4.19)	**0.046**	7.40(1.25-43-67)	**0.027**
**Was pregnancy planned**						
Yes	71(56.4)	77(48.7)	1			
No	55(43.7)	81(51.3)	1.36(0.85-2.17)	**0.200**	0.54(0.23-1.23)	0.147
**Relationship level**						

**Age of partner (years)**						
18-28	35 (27.8)	57(36.1)	1			
29-39	54(42.9)	78(49.4)	0.89(0.51- 1.53)	0.666	1.07(0.42-2.71)	0.880
40-49	22(17.5)	22(13.9)	0.61(0.30-1.27)	0.188	0.22(0.05-0.94)	**0.041**
>49 years	15(11.9)	1(0.6)	0.04(0.01- 0.32)	**0.002**	0.01(0.001-0.112)	**<.001**
**Education level of partner**						
No education	61(48.4)	100(63.3)	1			
Primary level	18(14.3)	30(19.0)	1.02(0.52-1.98)	0.961	0.87(0.34-2.25)	0.781
Post-primary	47(37.3)	28(17.7)	0.36(0.21-0.64)	**<.001**	0.23(0.08-0.64)	**0.005**
**Partner employed**						
Yes	71(56.3)	74(47.4)	1			
No	56(43.7)	47(52.6)	1.43(0.89-2.29)	**0.137**	2.82(1.28-6.17)	**0.010**
**Duration in the relationship**						
< 5 years	53 (42.1)	101 (63.9)	1			
> 5years	73 (57.9)	57 (36.1)	0.41 (0.25-0.66)	**<.001**	0.38(0.11-1.33)	0.130
Have you ever experienced IPSV when not pregnant?						
No	115(91.3)	92(58.2)	1			
Yes	11(8.7)	66(41.8)	2.66(3.74-5.02)	**<.001**	11.06(3.77-35.72)	**<.001**
**Level communication with partner**						
High	56(44.4)	34(21.8)				
Moderate	63(50.0)	106(68.0)	2.77(1.63- 4.70)	**<.001**	2.83(1.32-6.04)	**0.006**
Low	7(5.6)	16(10.2)	3.76(1.41-0.08)	**0.008**	0.38(0.06-2.26)	0.288
**Partner involvement in gambling**						
Yes	15(11.9)	38(24.1)	1			
No	111(88.1)	120(75.9)	0.43(0.22-0.81)	**0.010**	0.44(0.17-1.20)	0.111
**Partner allows visits to friends and neighbors**						
Yes	120(95.3)	144(91.1)	1			
No	6(4.8)	14(8.9)	1.94(o.72-5.21)	**0.186**	0.76(0.13-4.34)	0.761
**Societal level**						
**Partner has gender preferences for children**						
Yes	14(11.1)	29(18.4)	1			
No	112(88.9)	129(81.7)	0.55(0.28-1.10)	**0.094**	omitted	

**Gender preference(n=43)**						
Female	10(71.4)	9(31.0)	1			
Male	4(28.6)	20(69.0)	5.56(1.36-22.56)	**0.016**	5.56(1.36-22.56)	**0.016**
**Marital rape is not considered criminal**						
No	61(48.4)	59(37.3)	1			
Yes	65(51.6)	99(62.7)	1.57(0.98-2.53)	**0.061**	1.38(0.83-2.29)	0.216
**Does anyone provide emotional support?**						
No	30(23.8)	21(13.3)	1			
Yes	96(76.2)	137(86.7)	2.04(1.10-3.77)	0.023	1.80(0.94-3.46)	0.077
A pregnant woman sexually abused is not allowed to report						
No	26(20.6)	18(11.4)				
Yes	100(79.4)	140(88.6)	2.02(1.05-3.89)	**0.035**	1.93(0.95-3.89)	0.065

Multivariate logistic regression analysis of the correlates of IPSV

Women in their second pregnancy had a 21.5% lower prevalence of IPSV (aPR=0.785, 95%CI: 0.684-0.901, p=0.029) than those in their third or subsequent pregnancy. Similarly, women who had one pregnancy (para-one or primipara) had a 25% lower prevalence of IPSV (APR=0.750, 95% CI: 0.571-0.985, p=0.039) than women who had none (nulliparity). More, women who rated their communication with their partners as high had a 45% lower prevalence of IPSV (aPR=0.555, 95%CI: 0.379-0.810, p=0.002) than those who rated it as low. Women in relationships lasting less than five years, on the other hand, had a 1.53 times higher prevalence of IPSV when compared to those in relationships lasting more than five years. IPSV prevalence decreased with increasing age of the spouse from 18 to 28 years (aPR=9.94, 95%CI: 1.477-66.85, p=0.018); 29 to 39 years (aPR=9.385, 95%CI: 1.398-14.963, p=0.021); and 40 to 49 years (aPR=7.954, 95%CI:1.165-54.286, p=0.034) compared to older spouses over 49 years. Also, spouses with a higher level of

education (secondary) had a 30% lower prevalence of IPSV (aPR=0.669, 95%CI: 0.537-0.833, p=0.001), while women with a lower level of education (primary) had a 2.93 higher prevalence of IPSV (aPR=2.927, 95%CI: 1.211-7.075, p=0.017) than those with a higher level of education (secondary), as shown in table 4~~3~~.

Table ~~3~~4 showing the multivariable logistic regression analysis of IPSV among ~~respondents~~pregnant women in Napak district, Uganda.

Variable	Pregnancy IPSV		cPR (CI at 95%)	P value	aPR (CI at 95%)	P value
	Experienced n = 158	Not experienced n = 126				
**Gravidity**						
One	33(62.3%)	20(37.7%)	1.325 (0.986 - 1.780)	0.062	0.813 (0.703 - 0.941)	0.066
Two	47(65.3%)	25(34.7%)	1.389 (1.063 - 1.815)	**0.016**	0.785 (0.684 - 0.901)	**0.029**
Three	31(52.5%)	28(47.5%)	1.118 (0.812 - 1.539)	0.494	0.894 (0.787 - 1.015)	0.498
More than three	47(47.0%)	53(53.0%)	1.000		1.000	
**Parity**						
One	41(51.9%)	38(48.1%)	0.753 (0.572 - 0.989)	**0.042**	0.750 (0.571 -0.985)	**0.039**
Two	34(60.7%)	22(39.3%)	0.880 (0.670 - 1.156)	0.359	0.874 (0.658 - 1.161)	0.353
Three	14(66.7%)	7(33.3%)	0.967 (0.682 - 1.369)	0.849	0.980 (0.689 - 1.393)	0.910
More than three	29(41.4%)	41(58.6%)	0.601 (0.433 - 0.834)	**0.002**	0.599 (0.426 - 0.843)	**0.003**
None	40(69.0%)	18(31.0%)	1.000		1.000	
**Level of communication with spouse**						
High	34(37.8%)	56(62.2%)	0.543 (0.372 - 0.793)	**0.002**	0.555 (0.379 - 0.810)	**0.002**
Moderate	106(62.7%)	63(37.3%)	0.902 (0.672 - 1.210)	0.490	0.926 (0.687 - 1.249)	0.616
Low	16(69.6%)	7(30.4%)	1.000		1.000	
**Duration in marital relationship**						
Less than five years	101(65.6%)	53(34.4%)	1.496 (1.194 - 1.874)	**<0.001**	1.533 (1.210 - 1.941)	**<0.001**
More than five years	57(43.8%)	73(56.2%)	1.000		1.000	
**Age range of spouse**						
Between 18 and 28 years	57(62.0%)	35(38.0%)	9.913 (1.476 - 66.574)	**0.018**	9.944 (1.477 - 66.950)	**0.018**
Between 29 and 39 years	78(59.1%)	54(40.9%)	9.455 (1.410 - 63.404)	**0.021**	9.383 (1.398 - 14.963)	**0.021**
Between 40 and 49 years	22(50.0%)	22(50.0%)	8.000 (1.172 - 54.600)	**0.034**	7.954 (1.165 - 54.286)	**0.034**
More than 49 years	1(6.3%)	15(93.8%)	1.000		1.000	
**Spouse formally educated**						
Yes	58(47.2%)	65(52.8%)	0.759 (0.608 - 0.948)	**0.015**	0.669 (0.537 - 0.833)	**<0.001**

No	100(62.1%)	61(37.9%)	1.000		1.000	
**Level of education of woman**						
Primary	30(62.5%)	18(37.5%)	2.656 (1.097 - 6.433)	**0.030**	2.927 (1.211 - 7.075)	**0.017**
Secondary	24(41.4%)	34(58.6%	1.759 (0.708 - 4.369)	0.224	1.662 (0.668 - 4.134)	0.274
Post-secondary	4(23.5%)	13(76.5%)	1.000		1.000	

Discussion

IPSV arguably poses a subtle challenge of reproductive health as it attributes towards various life-threatening complications, namely; postpartum hemorrhage, sepsis, complications of childbirth, and fetomaternal mortality (2). In this study, the prevalence of IPSV was 56% (n = 158), and this was higher compared to most previous studies, for example 40.8% reported from northern Ethiopia (16), 19.9% in western Ethiopia (28~~7~~), 44.6% in Nigeria (29~~8~~), 18.8% in Tanzania (30~~29~~), 9.2% (31~~0~~) and 35.3% (32~~1~~) in Kenya, and 10.6% reported from a previous Ugandan study (18). On the other hand, the obtained prevalence is lower than the 70.3% reported from southwestern Uganda (19). The variations in the reported IPSV attests to its growing and disproportionate burden for varied ethnicities. Contrasting to majority of the studies, this study considered IPSV as defined by the World Health Organization, which includes rape, unwanted sexual advances, denial to use contraception, forced abortion, or denial to use protective measures against sexually transmitted infections (STIs) (5). Also, the studied population is highly patriarchal, and this socio-traditional fabric may as well account for the observed high IPSV prevalence. This suggests an urgent need for concerted ethnical approaches to avert the socio-cultural dynamics that negates reproductive health outcomes.

The study indicated that the prevalent form of IPSV was unwanted sexual advances (N=101~~0~~, 35.6~~63.3~~%), a finding that may suggest no physical, but rather emotional violation. Consistent to

previous studies, the associated violence may complicate the gestational process as well as augmenting the parturition (3,4,6,7).

As the prevalence of IPSV was higher in multigravidas, both the physiological and anatomic variances may be considered. It seems multigravidas tend to experience more pregnancy-associated mood changes, and may impair sexual drive. Premised on this, multigravidas tend to be less receptive of their spouse's sexual advances and are thus martially raped. Also, whereas sexual drive reduces in the first and third trimesters among all women; the effect is more exacerbated among multigravida women as they additionally experience other pregnancy complications (332). Relationship for less than five years implied that perhaps the couple had yet cultivated healthy communication between them, which makes dialogue less likely, thus increasing the risk of IPSV. This is consistent with previous reports (343, 354), and emphasizes relationship gaps due to breakdown in communication and negotiations. The age of the spouse was significantly correlated with IPSV, consistent with previous reports (365-387). Young age translated to more IPSV, and this may relate both to hormonal and behavioral attributes. Also, with unemployability and emerging drug as well as substance abuses, it is plausible to find a high IPSV in this age range. From this study, education lessened the risk of IPSV. Consistent with other studies (343, 376), formal education comes with numerous positive attributes including being employed and also possible knowledge about the effects of IPSV. Generally, educated men tend to have relatively less controlling behavior due to the limited or non-embracement of patriarchy and male dominance.

The findings of this study ought to be interpreted in light of some limitations. IPSV was self-reported and this depends on the recall and respondents ability to disclose a rather confidential marital prospect. There are population-specific traditional norms which may affect the study

findings. The study did not qualitatively explore on the practice of IPSV, and this definitely leaves a knowledge gap that requires critical attention to eliminate the IPSV practice. Also, this study may be limited by the interviewer bias and social desirability responses. More, this study did report on partners'/men's characteristics such as their involvement in risky behavior like alcohol/drug abuse, which would be of great value to understanding the IPSV practice and inform policy.

Conclusion

This study reports a high prevalence of IPSV among pregnant women and it was associated with women being in their third or subsequent pregnancies, nulliparity, poor-communication with a partner, being in relationship for less than five years, lower age of the spouse, and a lower level of education. Based on the findings, it is important to sensitize the public on the dangers of IPSV to avert the practice are required. A multipronged approach of health education and male involvement in addressing the IPSV practice are desired.

Acknowledgement

We are grateful to the study participants and research assistants.

References

1. UN Women. Ending violence against women. 2022 https://www.unwomen.org/en/what-we-do/ending-violence-against-women (Accessed June 18, 2023)
2. United Nations. Gender-based violence against women and girls. 2022 Available from https://www.ohchr.org/en/women/gender-based-violence-against-women-and-girls (Accessed August 24, 2023)
3. Clephane K, Wilson MC, Heiman JR, Craig AN, Lorenz T. Sexual violence history predicts changes in vaginal immune parameters during sexual arousal. Brain, behavior, and immunity. 2022 Aug 1;104:171-80.

4. Lohner L, Nigbur L, Klasen C, Witzel I, Garland J, Ondruschka B, Anders S. Vaginal injuries after consensual sexual intercourse—a survey among office-based gynecologists in Hamburg, Germany. Forensic Science, Medicine and Pathology. 2022 Sep;18(3):352-8.
5. WHO. (2021). Violence against women. Available from https://www.who.int/news-room/fact-sheets/detail/violence-against-women
6. Fekadu Dadi A, Miller ER, Mwanri L. Antenatal depression and its association with adverse birth outcomes in low and middle-income countries: a systematic review and meta-analysis. PloS one. 2020 Jan 10;15(1):e0227323.
7. Govender D, Naidoo S, Taylor M. Antenatal and postpartum depression: prevalence and associated risk factors among adolescents' in KwaZulu-Natal, South Africa. Depression research and treatment. 2020 Oct;2020.
8. Hassan MF, Rund N, El-Tohamy O, Moussa M, Ali YZ, Moussa N, Abdelrazik AA, Abdallah EA. Does aerobic vaginitis have adverse pregnancy outcomes? prospective observational study. Infectious diseases in obstetrics and gynecology. 2020 Jan 18;2020.
9. Cerveny L, Murthi P, Staud F. HIV in pregnancy: Mother-to-child transmission, pharmacotherapy, and toxicity. Biochimica et Biophysica Acta (BBA)-Molecular Basis of Disease. 2021 Oct 1;1867(10):166206.
10. Torres-de la Roche LA, Krentel H, Devassy R, de Wilde MS, Leicher L, De Wilde RL. Surgical repair of genital injuries after sexual abuse. GMS Interdisciplinary plastic and reconstructive surgery DGPW. 2019;8.
11. Selai C, Elmalem MS, Chartier-Kastler E, Sassoon N, Hewitt S, Rocha MF, Klitsinari L, Panicker JN. Systematic review exploring the relationship between sexual abuse and lower urinary tract symptoms. International Urogynecology Journal. 2023 Mar;34(3):635-53.
12. Agarwal S, Prasad R, Mantri S, Chandrakar R, Gupta S, Babhulkar V, Srivastav S, Jaiswal A, Wanjari MB. A comprehensive review of intimate partner violence during pregnancy and its adverse effects on maternal and fetal health. Cureus. 2023 May 20;15(5).
13. Kalunga K, Ilunga-Mbaya E, Maroy R, Jonathan YT, Olivier NN. Maternal-Fetal Pregnancy Complications of in Minors Raped with or without Physical Restraint in Eastern Democratic Republic of Congo. Open Journal of Obstetrics and Gynecology. 2023 Jan 16;13(1):1-2.
14. U.S. Department of Health & Human Services. Laws on violence against women. 2018 Accessed on 2/10/2020 from https://www.womenshealth.gov/relationships-and-safety/get-help/laws-violence-against-women
15. World Bank. Ending Violence Against Women And Girls: Global And Regional Trends In Women's Legal Protection Against Domestic Violence And Sexual Harassment. 2018. Accessed from http://pubdocs.worldbank.org/en/140781519943384134/EndingViolenceAgainstWomenandGirlsGBVLawsFeb2018.pdf
16. Berhanie E, Gebregziabher D, Berihu H, Gerezgiher A, Kidane G. Intimate partner violence during pregnancy and adverse birth outcomes: a case-control study. Reproductive health. 2019 Dec;16(1):1-9.
17. Rasch V, Van TN, Nguyen HT, Manongi R, Mushi D, Meyrowitsch DW, Gammeltoft T, Wu CS. Intimate partner violence (IPV): the validity of an IPV screening instrument utilized among pregnant women in Tanzania and Vietnam. PloS one. 2018 Feb 1;13(2):e0190856.

18. Epuitai J, Udho S, Auma AG, Nabirye RC. Intimate partner violence among pregnant women in Uganda. African Journal of Midwifery and Women's Health. 2019 Apr 2;13(2):1-5.

19. Katushabe E, Asiimwe JB, Batwala V. Intimate partner violence disclosure and associated factors among pregnant women attending a city hospital in South-Western Uganda: a cross-sectional study. BMC pregnancy and childbirth. 2022 Jun 13;22(1):484.

20. Achen S, Rwabukwali CB, Atekyereza P. Socio-cultural perceptions of sexuality influencing the sexual and reproductive health of pastoral adolescent girls in Karamoja sub-region in Uganda. Social Sciences & Humanities Open. 2021 Jan 1;4(1):100191.

21. Park YS, Konge L, Artino AR. The positivism paradigm of research. Academic medicine. 2020 May 1;95(5):690-4.

~~22. Villar J, Bergsjo PW. WHO antenatal care randomized trial: manual for the implementation of the new model. InWHO antenatal care randomized trial: manual for the implementation of the new model 2002 (pp. 37-37).~~

22. Tunçalp Ö, Pena-Rosas JP, Lawrie T, Bucagu M, Oladapo OT, Portela A, Gülmezoglu AM. WHO recommendations on antenatal care for a positive pregnancy experience-going beyond survival. Bjog. 2017 May 1;124(6):860-2.

23. Mugisha C, Odit A, Luzze H. Uganda Clinical Guidelines: National Guidelines on Management of Common Conditions. Uganda: Ministry of Health. 2010:325-78.

24. Daniel W. Biostatistics: A Foundation for analysis in the health sciences, 7th edR Wiley. New York. 1999;141(2).

~~24.~~25. World Health Organization. Understanding and addressing violence against women: Intimate partner violence. World Health Organization; 2012.

~~25.~~26. Kafka JM, Moracco KE, Young BR, Taheri C, Graham LM, Macy RJ, Proescholdbell SK. Fatalities related to intimate partner violence: towards a comprehensive perspective. Injury prevention. 2021 Apr 1;27(2):137-44.

~~26.~~27. Martinez BA, Leotti VB, Silva GD, Nunes LN, Machado G, Corbellini LG. Odds ratio or prevalence ratio? An overview of reported statistical methods and appropriateness of interpretations in cross-sectional studies with dichotomous outcomes in veterinary medicine. Frontiers in veterinary science. 2017 Nov 10;4:193.

~~27.~~28. Abebe Abate B, Admassu Wossen B, Tilahun Degfie T. Determinants of intimate partner violence during pregnancy among married women in Abay Chomen district, Western Ethiopia: a community based cross sectional study. BMC women's health. 2016 Dec;16(1):1-8.

~~28.~~29. Orpin J, Papadopoulos C, Puthussery S. The prevalence of domestic violence among pregnant women in Nigeria: a systematic review. Trauma, Violence, & Abuse. 2020 Jan;21(1):3-15.

~~29.~~30. Mahenge B, Stöckl H, Abubakari A, Mbwambo J, Jahn A. Physical, sexual, emotional and economic intimate partner violence and controlling behaviors during pregnancy and postpartum among women in Dar es Salaam, Tanzania. PloS one. 2016 Oct 18;11(10):e0164376.

~~30.~~31. Stiller M, Bärnighausen T, Wilson ML. Intimate partner violence among pregnant women in Kenya: forms, perpetrators and associations. BMC women's health. 2022 Dec;22(1):1-25.

~~31.~~32. Onuonga K, Onguru D, Ogolla S, Mutanyi JA. Intimate partner violence among pregnant women attending prenatal care in Bondo sub- county, Western Kenya. International Journal of Gynecology & Obstetrics. 2023 Jan;160(1):106-12.

Formatted: Font color: Auto, Pattern: Clear

~~32.~~33. Mehari MA, Maeruf H, Robles CC, Woldemariam S, Adhena T, Mulugeta M, Haftu A, Hagose H, Kumsa H. Advanced maternal age pregnancy and its adverse obstetrical and perinatal outcomes in Ayder comprehensive specialized hospital, Northern Ethiopia, 2017: a comparative cross-sectional study. BMC pregnancy and childbirth. 2020 Dec;20(1):1-0.
~~33.~~34. Semahegn A, Mengistie B. Domestic violence against women and associated factors in Ethiopia; systematic review. Reproductive health. 2015 Dec;12(1):1-2.
~~34.~~35. Choi EY, Hyun HJ. A predictive model of domestic violence in multicultural families focusing on perpetrator. Asian nursing research. 2016 Sep 1;10(3):213-20.
~~35.~~36. Owaka IO, Nyanchoka MK, Atieli HE. Intimate partner violence in pregnancy among antenatal attendees at health facilities in West Pokot county, Kenya. Pan African Medical Journal. 2017;28(1).
~~36.~~37. Lencha B, Ameya G, Baresa G, Minda Z, Ganfure G. Intimate partner violence and its associated factors among pregnant women in Bale Zone, Southeast Ethiopia: A cross-sectional study. PloS one. 2019 May 1;14(5):e0214962.
~~37.~~38. Barnawi FH. Prevalence and risk factors of domestic violence against women attending a primary care center in Riyadh, Saudi Arabia. Journal of interpersonal violence. 2017 Apr;32(8):1171-86.

List of legends

Supplementary Material_Data Collection Tool

Supporting Information_Research and Ethics Committee Approval

Kampala, Uganda.
21st September 2023

Professor Muthusamy Sivakami
Academic Editor
PLOS Global Public Health

Dear Professor Muthusamy Sivakami

RE: Responses to the reviewers' comments for the submitted manuscript titled: "Prevalence and Correlates of Intimate Partner Sexual Violence among Pregnant Women in Napak District, Northeastern Uganda" Manuscript ID: PGPH-D-23-01429

We are grateful for the critical reviewers' comments, and we confirm to you that the changes have been effected in the revised document (R1), and these are indicated in the table below;

**Journal Requirements**	
**Comment**	**Response**
We have noticed that you have uploaded Supporting Information files, but you have not included a list of legends. Please add a full list of legends for your Supporting Information files after the references list.	A list of legends has been included after the reference section.
**Additional Editor Comments (if provided):**	
Kindly respond to our reviewers, especially reviewer 1. We appreciate your interest with PLOS-GPH.	Review comments have been addressed.
Have the authors made all data underlying the findings in their manuscript fully available (please refer to the Data Availability Statement at the start of the manuscript PDF file)?	

The PLOS Data policy requires authors to make all data underlying the findings described in their manuscript fully available without restriction, with rare exception. The data should be provided as part of the manuscript or its supporting information, or deposited to a public repository. For example, in addition to summary statistics, the data points behind means, medians and variance measures should be available. If there are restrictions on publicly sharing data—e.g. participant privacy or use of data from a third party—those must be specified.<br><br>Reviewer #1: No	A correction to avail all the data has been made. To this, a bivariate analysis table has been considered as table 3, and a multivariate analysis table named as table 4. Further, the data availability statement has been revised to remove any data limitations in accordance with the PLOS Data policy.
**Reviewers comments and their responses**	
**Reviewer comments**	**Response**
Reviewer #1: Great work. This is a very important subject and will definitely contribute to prevention of IPSV.<br>It could even be better by attending to reviewer comments. Please do not assume that the your reader will understands everything in the methods section. There are parts that are a bit unclear and need to be clarified to improve the quality of this paper.	The authors appreciates your compliment, and have revised the manuscript as guided by the review comments.
**Introduction**<br>Line 55. Please rewrite "……, particularly SDG 5 target 2."<br><br>Line 63. Please write STIs in full when using it for the first time	Line 55; "…has been re-written…particularly SDG 5 target 2".<br><br>Line 63; STIs has been written in full as sexually transmitted infections.

Line 64. Vertical transmission of what? Please make this sensible<br><br>Lines 93-98. Please state clearly this is an old WHO recommendation for Antenatal care or focused ANC. The 2016 WHO ANC recommendation is the most current and requires 8 ANC visits.	Line 64; Vertical transmission …has changed to: "…vertical transmission of Human Immunodeficiency Virus (HIV) from the mother to the unborn baby" to make it more sensible.<br><br>Lines 94-96; a revision to reflect the recommendation of the eight antenatal care contacts with health system during each pregnancy has been added, and reference number 22 has been updated to reflect this recommendation.
**Methods**<br>Line 100-101. "The presumed that within the studied period, ……". This part does not make sense here please take it to data collection or sample section.<br><br>Line 108. Please rewrite to make meaningful, "……………….by the majority of pregnant…"<br><br>Line 116. Please add "representative sample". Please state which type of bias this minimized.<br><br>Lines 117-123. Data collection tool and approach. This section is good but leaves a lot of important information out. It is known that some women come to the hospitals with their husbands. How was data collected in the presence of their husbands/ How confidential was the data collection process. Are all the women who were sampled were interviewed in the absence of their husbands. When were they interviewed? Where were they interviewed? At the hospital? If so was	lines 112-114 ; This part has been moved to lines 112-114 of the sampling procedures<br><br>Line 110;….suggested improvements have included….." by the majority of the pregnant women…"<br><br>Line 120; …the "representative sample" has been added and the type of bias minimized has been specified.<br><br>Lines 124 – 127; The important details of data collection have been included. Specifically, it is now indicated that: "The interviews were conducted on a one-on-one basis, with husbands excluded from participating to ensure candid responses. These interviews took place in a health facility setting during the ANC visits of eligible women with dedicated rooms provided to ensure privacy and confidentiality".

there a designated room for this interview? How was the privacy achieved	Line 127; A correction has been made.
Line 120. Please correct "…. A pilot testing of…….	Line 132……phrase added as suggested..
Line 125. Please add "The dependent………was experience of IPSV during pregnancy,….."	
Lines 128-131. Please state and reference the guideline(s) you have used to formulate these questions. A statement such as "A woman was said to have been sexually violated by her intimate partner if she is reported / having had her spouse make sexual advances to her when she was not interested" is vague, it assumes the man already knows whether or not his pregnant wife is interested or not. Making sexual advances to your spouse should not be assumed as violence unless the male partner persists after the women indicates she is not interested, then it is violation. Even women make sexual advances towards their husbands and it is not considered sexual violence unless she persists when he says no -Unusual case. But please make complete the statement to make it clear and not misleading to the reader.	More definitive information has been added, and a reference (number 25) has been provided. As well, the reference list has been updated accordingly all through the manuscript.<br><br>Also, a correction in lines 138-145 has been made. The corrected paragraph reads: "A woman was said to have been sexually violated by her intimate partner if she reported/having had her spouse make sexual advances to her when she indicated that she was not interested, forced to have sexual intercourse when she did not want to (rape), or denied the use STI preventive measures in the past 2 or 8 months. According to WHO (25), sexual advances made by an intimate partner without the consent or against the will of the other person is a form of intimate partner violence".
Line 155-156. How did you reach this cut-off to take only those that were statistically significant P<0.05? Assume a variable was 0.057, wouldn´t this indicate something that warrant further investigations or inclusion in further analysis. Why not use the commonly used P<0.2 or P>0.25	Lines 166-167; A revision to clarify the bivariate analysis has been made for clarity. It is now indicated that: "At bivariate analyses, variables whose p-value were ≤0.2 were considered". Because of this change, a bivariate table (now named as table 3) has been considered (lines 205 to lines 210) in the results section. Due to this change, the previous table 3

cut-off for selection of variables to be admitted to the multivariate logistic model?	(multivariate analysis table) has now been named as table 4.
Line 157-159. Please state clearly when you removed or added the independent variables. It is not enough to say……. model was having a P<0.05.	A correction to the statistical analysis has been made to indicate that: "Bivariate analysis was carried out with independent variables to select the variables for multivariable analysis at a p-value ≤0.20".<br><br>Also, "Multivariate analysis was then considered using the log-binomial model in which all variables that were found to be statistically significant at bivariate analysis were fitted. We identified the correlates of IPSV stopping when all the remaining variables were significant at a 5% (0.05) level of significance. Backward stepwise Likelihood Ratio (LR) was used to find the significant predictors of IPSV among the pregnant women. Statistical significance at this point was set at 5% (p<0.05), and the findings were reported using adjusted prevalence ratios along with their corresponding confidence intervals at 95%".
All the table title are all incomplete. Please complete them by adding the "in Napak district, Uganda" or among pregnant women (respondents) in Napak district, Uganda.	A correction to tables 1, 2, 3 and 4 has been made to indicate that it was in Napak district, Uganda.
Lines 247-251. Please add other limitations related to the interview privacy, interviewer bias, social desirability responses if present e.t.c. that could have biased the study	Lines 277-280; It has been indicated that: "Also, this study may be limited by the interviewer bias and social desirability responses".
Reviewer #2:<br>A good attempt by the authors. The issue of IPSV during pregnancy is a very serious and important RH topic, particularly in a society or sub-	The authors are grateful of the reviewer compliments and further emphasizing the importance of this topic.

population experiencing such high prevalence (56%). However, the paper could have elaborated on the forms of 'unwanted sexual advances' and circumstances leading to 'rape or forced sexual intercourse'. Some may argue that the partners/men are unaware of their partners' pregnancy status (first trimester, etc) or ignorant of reproductive rights. Inclusion of some of partners'/men's characteristics in the analysis (if data permits; e.g. their age, education, economic status, risky behaviours like alcohol/drug abuse, gambling, etc), which could be of great value to policy and our understanding of such issue.<br><br>Authors are advised to cross-check the prevalent values (%) mention in the text with that in the relevant tables. For instance, in Discussion section, page 12 (last para; lines 226-227), on unwanted sexual advances the authors wrote, N=100, 63.3%, which is different from the values and number in the Table 2 and in the text in page 9, line 187	Inclusion of some of the partners/men characteristics in the analysis has considered as seen in the bivariate table (table 3). However, some characteristics such as their risky behavior like alcohol/drug abuse, which would be of great value to understanding the IPSV practice and inform policy.<br><br>A careful check of the prevalent value has been made and a change made accordingly.

We are grateful to all the reviewers' comments, and we confirm that all changes are indicated in track change of the R1 manuscript. Thank you so much for your guidance and support to improve this manuscript. We look forward to acceptance of this manuscript and publication.

Sincerely,

Ivan Mugisha Taremwa
+256774346368
imugisha@ymail.com
