# Supplementary material for: Prevalence and correlates of intimate partner sexual violence among pregnant women in Napak district, Northeastern Uganda
Source: PLOS Glob Public Health. 2024 Feb 1;4(2):e0002286. doi: 10.1371/journal.pgph.0002286 (PMC10833580; doi:10.1371/journal.pgph.0002286)
Supplement: Attachment — Submitted filename: Response to reviewers comments.docx [file pgph.0002286.s003.docx]

Kampala, Uganda.

21^st^ September 2023

Professor Muthusamy Sivakami
Academic Editor
PLOS Global Public Health

Dear Professor Muthusamy Sivakami

**RE: Responses to the reviewers' comments for the submitted manuscript titled: “Prevalence and Correlates of Intimate Partner Sexual Violence among Pregnant Women in Napak District, Northeastern Uganda” Manuscript ID: PGPH-D-23-01429**

We are grateful for the critical reviewers’ comments, and we confirm to you that the changes have been effected in the revised document (R1), and these are indicated in the table below;

| **Journal Requirements** | |
| --- | --- |
| **Comment** | **Response** |
| We have noticed that you have uploaded Supporting Information files, but you have not included a list of legends. Please add a full list of legends for your Supporting Information files after the references list. | A list of legends has been included after the reference section. |
| **Additional Editor Comments (if provided):** |  |
| Kindly respond to our reviewers, especially reviewer 1. We appreciate your interest with PLOS-GPH. | Review comments have been addressed. |
| Have the authors made all data underlying the findings in their manuscript fully available (please refer to the Data Availability Statement at the start of the manuscript PDF file)?  The [PLOS Data policy](https://journals.plos.org/globalpublichealth/s/data-availability) requires authors to make all data underlying the findings described in their manuscript fully available without restriction, with rare exception. The data should be provided as part of the manuscript or its supporting information, or deposited to a public repository. For example, in addition to summary statistics, the data points behind means, medians and variance measures should be available. If there are restrictions on publicly sharing data—e.g. participant privacy or use of data from a third party—those must be specified.  Reviewer #1: No | A correction to avail all the data has been made. To this, a bivariate analysis table has been considered as table 3, and a multivariate analysis table named as table 4. Further, the data availability statement has been revised to remove any data limitations in accordance with the PLOS Data policy. |
| **Reviewers comments and their responses** | |
| **Reviewer comments** | **Response** |
| Reviewer #1: Great work. This is a very important subject and will definitely contribute to prevention of IPSV. It could even be better by attending to reviewer comments. Please do not assume that the your reader will understands everything in the methods section. There are parts that are a bit unclear and need to be clarified to improve the quality of this paper. | The authors appreciates your compliment, and have revised the manuscript as guided by the review comments. |
| **Introduction**  Line 55. Please rewrite “……, particularly SDG 5 target 2.”  Line 63. Please write STIs in full when using it for the first time  Line 64. Vertical transmission of what? Please make this sensible  Lines 93-98. Please state clearly this is an old WHO recommendation for Antenatal care or focused ANC. The 2016 WHO ANC recommendation is the most current and requires 8 ANC visits. | Line 55; “…has been re-written…particularly SDG 5 target 2”.  Line 63; STIs has been written in full as sexually transmitted infections.  Line 64; Vertical transmission …has changed to: “…vertical transmission of Human Immunodeficiency Virus (HIV) from the mother to the unborn baby” to make it more sensible.    Lines 94-96; a revision to reflect the recommendation of the eight antenatal care contacts with health system during each pregnancy has been added, and reference number 22 has been updated to reflect this recommendation. |
| **Methods**  Line 100-101. “The presumed that within the studied period, ……”. This part does not make sense here please take it to data collection or sample section.  Line 108. Please rewrite to make meaningful, “………………..by the majority of pregnant…”  Line 116. Please add “representative sample”. Please state which type of bias this minimized.  Lines 117-123. Data collection tool and approach.  This section is good but leaves a lot of important information out. It is known that some women come to the hospitals with their husbands. How was data collected in the presence of their husbands/ How confidential was the data collection process. Are all the women who were sampled were interviewed in the absence of their husbands. When were they interviewed? Where were they interviewed? At the hospital? If so was there a designated room for this interview? How was the privacy achieved  Line 120. Please correct “…. A pilot testing of…….  Line 125. Please add “The dependent………was experience of IPSV during pregnancy,…..”  Lines 128-131. Please state and reference the guideline(s) you have used to formulate these questions. A statement such as “A woman was said to have been sexually violated by her intimate partner if she is reported / having had her spouse make sexual advances to her when she was not interested” is vague, it assumes the man already knows whether or not his pregnant wife is interested or not. Making sexual advances to your spouse should not be assumed as violence unless the male partner persists after the women indicates she is not interested, then it is violation. Even women make sexual advances towards their husbands and it is not considered sexual violence unless she persists when he says no -Unusual case. But please make complete the statement to make it clear and not misleading to the reader.  Line 155-156. How did you reach this cut-off to take only those that were statistically significant P<0.05? Assume a variable was 0.057, wouldn´t this indicate something that warrant further investigations or inclusion in further analysis. Why not use the commonly used P<0.2 or P>0.25 cut-off for selection of variables to be admitted to the multivariate logistic model? | lines 112-114 ; This part has been moved to lines 112-114 of the sampling procedures  Line 110;….suggested improvements have included…..” by the majority of the pregnant women…”  Line 120; …the “representative sample” has been added and the type of bias minimized has been specified.  Lines 124 – 127; The important details of data collection have been included. Specifically, it is now indicated that: “The interviews were conducted on a one-on-one basis, with husbands excluded from participating to ensure candid responses. These interviews took place in a health facility setting during the ANC visits of eligible women with dedicated rooms provided to ensure privacy and confidentiality”.  Line 127; A correction has been made.  Line 132……phrase added as suggested..  More definitive information has been added, and a reference (number 25) has been provided. As well, the reference list has been updated accordingly all through the manuscript.  Also, a correction in lines 138-145 has been made. The corrected paragraph reads: “A woman was said to have been sexually violated by her intimate partner if she reported/having had her spouse make sexual advances to her when she indicated that she was not interested, forced to have sexual intercourse when she did not want to (rape), or denied the use STI preventive measures in the past 2 or 8 months. According to WHO (25), sexual advances made by an intimate partner without the consent or against the will of the other person is a form of intimate partner violence”.  Lines 166-167; A revision to clarify the bivariate analysis has been made for clarity. It is now indicated that: “At bivariate analyses, variables whose p-value were ≤0.2 were considered”. Because of this change, a bivariate table (now named as table 3) has been considered (lines 205 to lines 210) in the results section. Due to this change, the previous table 3 (multivariate analysis table) has now been named as table 4. |
| Line 157-159. Please state clearly when you removed or added the independent variables. It is not enough to say……. model was having a P<0.05.  All the table title are all incomplete. Please complete them by adding the “in Napak district, Uganda” or among pregnant women (respondents) in Napak district, Uganda. | A correction to the statistical analysis has been made to indicate that: “Bivariate analysis was carried out with independent variables to select the variables for multivariable analysis at a p-value ≤0.20”.  Also, “Multivariate analysis was then considered using the log-binomial model in which all variables that were found to be statistically significant at bivariate analysis were fitted. We identified the correlates of IPSV stopping when all the remaining variables were significant at a 5% (0.05) level of significance. Backward stepwise Likelihood Ratio (LR) was used to find the significant predictors of IPSV among the pregnant women. Statistical significance at this point was set at 5% (p<0.05), and the findings were reported using adjusted prevalence ratios along with their corresponding confidence intervals at 95%”.  A correction to tables 1, 2, 3 and 4 has been made to indicate that it was in Napak district, Uganda. |
| Lines 247-251. Please add other limitations related to the interview privacy, interviewer bias, social desirability responses if present e.t.c. that could have biased the study | Lines 277-280; It has been indicated that: “Also, this study may be limited by the interviewer bias and social desirability responses”. |
| Reviewer #2:  A good attempt by the authors. The issue of IPSV during pregnancy is a very serious and important RH topic, particularly in a society or sub-population experiencing such high prevalence (56%). However, the paper could have elaborated on the forms of 'unwanted sexual advances' and circumstances leading to 'rape or forced sexual intercourse'. Some may argue that the partners/men are unaware of their partners' pregnancy status (first trimester, etc) or ignorant of reproductive rights. Inclusion of some of partners'/men's characteristics in the analysis (if data permits; e.g. their age, education, economic status, risky behaviours like alcohol/drug abuse, gambling, etc), which could be of great value to policy and our understanding of such issue.  Authors are advised to cross-check the prevalent values (%) mention in the text with that in the relevant tables. For instance, in Discussion section, page 12 (last para; lines 226-227), on unwanted sexual advances the authors wrote, N=100, 63.3%, which is different from the values and number in the Table 2 and in the text in page 9, line 187 | The authors are grateful of the reviewer compliments and further emphasizing the importance of this topic.  Inclusion of some of the partners/men characteristics in the analysis has considered as seen in the bivariate table (table 3). However, some characteristics such as their risky behavior like alcohol/drug abuse, which would be of great value to understanding the IPSV practice and inform policy.  A careful check of the prevalent value has been made and a change made accordingly. |

We are grateful to all the reviewers’ comments, and we confirm that all changes are indicated in track change of the R1 manuscript. Thank you so much for your guidance and support to improve this manuscript. We look forward to acceptance of this manuscript and publication.

Sincerely,

Ivan Mugisha Taremwa

+256774346368

[imugisha@ymail.com](mailto:imugisha@ymail.com)
