# Supplementary material for: Prevalence and correlates of intimate partner sexual violence among pregnant women in Napak district, Northeastern Uganda
Source: PLOS Glob Public Health. 2024 Feb 1;4(2):e0002286. doi: 10.1371/journal.pgph.0002286 (PMC10833580; doi:10.1371/journal.pgph.0002286)
Supplement: Attachment — Submitted filename: R2-Response to reviewers comments.docx [file pgph.0002286.s005.docx]

Kampala, Uganda.

20^th^ December 2023

Professor Muthusamy Sivakami
Academic Editor
PLOS Global Public Health

Dear Professor Muthusamy Sivakami

**RE: Responses to the reviewers' comments for the submitted manuscript titled: “Prevalence and Correlates of Intimate Partner Sexual Violence among Pregnant Women in Napak District, Northeastern Uganda” Manuscript ID: PGPH-D-23-01429**

We are grateful for the critical reviewers’ comments, and we confirm to you that the changes have been effected in the revised document (R2), and these are indicated in the table below;

| **Journal Requirements** | |
| --- | --- |
| **Comment** | **Response** |
| 1. If the authors have adequately addressed your comments raised in a previous round of review and you feel that this manuscript is now acceptable for publication, you may indicate that here to bypass the “Comments to the Author” section, enter your conflict of interest statement in the “Confidential to Editor” section, and submit your "Accept" recommendation. | Reviewer #3: (No Response) |
| 2. Does this manuscript meet PLOS Global Public Health’s [publication criteria](https://journals.plos.org/globalpublichealth/s/criteria-for-publication)? Is the manuscript technically sound, and do the data support the conclusions? The manuscript must describe methodologically and ethically rigorous research with conclusions that are appropriately drawn based on the data presented. | Reviewer #3: Yes |
| 3. Has the statistical analysis been performed appropriately and rigorously? | Reviewer #3: I don't know  **Author response**: We confirm that statistical analysis been performed appropriately and rigorously |
| Have the authors made all data underlying the findings in their manuscript fully available (please refer to the Data Availability Statement at the start of the manuscript PDF file)?  The [PLOS Data policy](https://journals.plos.org/globalpublichealth/s/data-availability) requires authors to make all data underlying the findings described in their manuscript fully available without restriction, with rare exception. The data should be provided as part of the manuscript or its supporting information, or deposited to a public repository. For example, in addition to summary statistics, the data points behind means, medians and variance measures should be available. If there are restrictions on publicly sharing data—e.g. participant privacy or use of data from a third party—those must be specified. | Reviewer #3: Yes |
| 5. Is the manuscript presented in an intelligible fashion and written in standard English?  PLOS Global Public Health does not copyedit accepted manuscripts, so the language in submitted articles must be clear, correct, and unambiguous. Any typographical or grammatical errors should be corrected at revision, so please note any specific errors here. | Reviewer #3: No  **Author response**: We confirm that language revision has been done to ensure there are no errors. The changes are indicated in track change. |
| **Review Comments to the Author** | |
| Reviewer #3: This issue is indeed a significant public health concern, and limited evidence is available globally.  Overall, writing needs to be improved. | |
| **Introduction** | |
| 1. It needs improvement, and the rationale for the study needs better articulation | An improvement in the manuscript has been made, and the rationale for the study has been in included in lines 76 to 79. |
| 2. In some places, too many things in a single sentence make it difficult to read and comprehend. | The sentences in the manuscript are revised to ensure that they are not lengthy. |
| **Method** | |
| 1. Sections need more clarity on the basis on which authors decided to select 3 out of 5 centres | As the study used a sampling approach of the available health centre IIIs, these (3) Health Centre 3’s were selected using a simple random sampling approach. this is shown in lines 112 to 113. |
| 2. Elaborate on the process of participant recruitment. | A section on participant enrollment has been included in the methods section and this is given in lines 111 to 118. |
| **Results** | |
| 1. The table 3 & 4 headings are not consistent with the type of analysis mentioned in the method section. | Table 3 shows the bivariate logistic regression analysis, while table 4 shows the multivariable logistic regression analysis of IPSV among pregnant women in Napak district, Uganda. The listed analyses has been revised, and it is now consistent with what is indicated in the methods section, lines 177 to 185. We hope you find this meaningful. |
| 2. In many places, the CI was set differently, e.g., 0.20 or 0.05. There should be a consistent way to report such information. | The CI was set at 80% for the bivariate analysis and 95% for the multivariable analysis. Also, consistent with this, we acknowledge that p-values were set at 0.20 for bivariate analysis, and 0.05 for multivariable analysis. The different p-values (0.20 and 0.05) are consistent with the statistical recommendations. we interpreted the final association based on the multivariable logistic regression at a p-value of ≤0.05, and a CI of 95%. We hope that this is clear to you. |
| 3. The interpretation of the results needs a careful approach. | We confirm that a careful consideration of the results has been considered, and we confirm that their interpretation aligns well with the statistical output. Variables with an association have been interpreted in light of the p-value (significant variables are those associated with a p-value of ≤0.20 for bivariate logistic regression, and ≤0.05 for multivariable logistic regression). We hope this is clear to you. |
| Discussion | |
| 1. The discussion part mentions so many things, but as a reader, it is most likely to get lost. For example, in many places, authors say that things are due to patriarchal systems and cultural norms. on the other hand, they also try to say that these are due to biological changes, etc. The reviewer is aware that there can be multiple explanations of the findings, but these should be sync and consistent. | We appreciate the reviewer’s views, and we agree that the patriarchal, culture and biological attributes are all contributors to the IPSV. This is consistent with the manuscript. |
| References: | |
| 1. A few references mentioned are not valid or accessible. For example, the WHO acts on IPSV. Another example is the prevalence of IPSV | 1. Reference number 5: “WHO. (2021). Violence against women.” is available at: <https://www.who.int/news-room/fact-sheets/detail/violence-against-women> . The site is available and can open. The definition given in this manuscript is in agreement with the definitions provided on the page. 2. Also, we have cross checked all the reference, and we confirm that they are available and accessible. We apologize if you are unable to access it on your side. |

We apologize for any inconveniences including failure of references to open your side when we can access them on our end.

We are grateful to all the reviewers’ comments, and we confirm that all changes are indicated in track change of the R2 manuscript. Thank you so much for your guidance and support to improve this manuscript. We look forward to acceptance of this manuscript and publication.

Sincerely,

Ivan Mugisha Taremwa

+256774346368

[imugisha@ymail.com](mailto:imugisha@ymail.com)
