# Supplementary material for: Prevalence and correlates of intimate partner sexual violence among pregnant women in Napak district, Northeastern Uganda
Source: PLOS Glob Public Health. 2024 Feb 1;4(2):e0002286. doi: 10.1371/journal.pgph.0002286 (PMC10833580; doi:10.1371/journal.pgph.0002286)
Supplement: Attachment — Submitted filename: Reviewer comments PGPH-D-23-01429.docx [file pgph.0002286.s002.docx]

**Prevalence and Correlates of Intimate Partner Sexual Violence 2 among Pregnant Women in Napak District, Northeastern Uganda**

**Reviewer comments**

This is an important work that highlights how IPSV affects maternal health and is important for policy improvements.

**Introduction**

Line 55. Please rewrite “……, particularly SDG 5 target 2.”

Line 63. Please write STIs in full when using it for the first time

Line 64. Vertical transmission of what? Please make this sensible

Lines 93-98. Please state clearly this is an old WHO recommendation for Antenatal care or focused ANC. The 2016 WHO ANC recommendation is the most current and requires 8 ANC visits.

Additionally, the statements “The gestation period was guided by the Ministry of Health, Uganda in adherence to 94 the World Health Organization recommendation of the four antenatal care visits” does not make sense. Perhaps you could say the ANC visits were guided by…..

**Methods**

Line 100-101. “The presumed that within the studied period, ……”. This part does not make sense here please take it to data collection or sample section.

Line 108. Please rewrite to make meaningful, “………………..by the majority of pregnant…”

Line 116. Please add “representative sample”. Please state which type of bias this minimized.

Lines 117-123. Data collection tool and approach.

This section is good but leaves a lot of important information out. It is known that some women come to the hospitals with their husbands. How was data collected in the presence of their husbands/ How confidential was the data collection process. Are all the women who were sampled were interviewed in the absence of their husbands. When were they interviewed? Where were they interviewed? At the hospital? If so was there a designated room for this interview? How was the privacy achieved?

Line 120. Please correct “…. A pilot testing of…….

Line 125. Please add “The dependent………was experience of IPSV during pregnancy,…..”

Lines 128-131. Please state and reference the guideline(s) you have used to formulate these questions. A statement such as “A woman was said to have been sexually violated by her intimate partner if she is reported / having had her spouse make sexual advances to her when she was not interested” is vague, it assumes the man already knows whether or not his pregnant wife is interested or not. Making sexual advances to your spouse should not be assumed as violence unless the male partner persists after the women indicates she is not interested, then it is violation. Even women make sexual advances towards their husbands and it is not considered sexual violence unless she persists when he says no -Unusual case. But please make complete the statement to make it clear and not misleading to the reader.

Line 155-156. How did you reach this cut-off to take only those that were statistically significant P<0.05? Assume a variable was 0.057, wouldn´t this indicate something that warrant further investigations or inclusion in further analysis. Why not use the commonly used P<0.2 or P>0.25 cut-off for selection of variables to be admitted to the multivariate logistic model?

Line 157-159. Please state clearly when you removed or added the independent variables. It is not enough to say……. model was having a P<0.05.

Results.

All the table title are all incomplete. Please complete them by adding the “in Napak district, Uganda” or among pregnant women (respondents) in Napak district, Uganda

Discussions

Lines 247-251. Please add other limitations related to the interview privacy, interviewer bias, social desirability responses if present e.t.c. that could have biased the study
